# Validation of a Motor Competence Assessment Tool for Children and Adolescents (KTK3+) With Normative Values for 6- to 19-Year-Olds

**DOI:** 10.3389/fphys.2021.652952

**Published:** 2021-06-23

**Authors:** Eline Coppens, Felien Laureys, Mireille Mostaert, Eva D'Hondt, Frederik J. A. Deconinck, Matthieu Lenoir

**Affiliations:** ^1^Department of Movement and Sports Sciences, Ghent University, Ghent, Belgium; ^2^Department of Movement and Sport Sciences, Vrije Universiteit Brussel, Brussels, Belgium

**Keywords:** coordination, motor development, motor proficiency, reference values, test battery, youth

## Abstract

The use of the short form of the Körperkoordinationstest für Kinder (KTK3) to evaluate children's and adolescents' motor competence (MC) is increasing. When combined with an alternating one-handed catching and throwing ball task, assessing eye-hand coordination (EHC), it has been shown that the different aspects of motor skills are adequately covered in one compact KTK3+ test battery, studied in 6- to 10-year-old children. The present study aimed to validate the KTK3+ test battery and to provide contemporary MC normative values for boys and girls from 6- to 19-year-olds. A total of 2,271 children and adolescents (1,112 boys, 1,159 girls) participated in this study and were evaluated on the four included test items: jumping sideways (JS), moving sideways (MS), balancing backwards (BB), supplemented by an EHC task. Children's participation in organised sport was registered using a demographic questionnaire. For the first objective, a factor analysis with multidimensional scaling demonstrated that the one-dimensional model provided the best fit, with all test items correlating to the same latent construct: “MC”. This was further supported with moderate to good correlations between all four test items (*r* = 0.453–0.799). Construct validity was investigated with a three-way MANOVA, demonstrating a significant multivariate interaction effect between sex and age group (*p* = 0.001) as well as a multivariate main effect of sex, age group, and organised sport participation (*p* < 0.001). Boys outperformed girls on two out of the four tests (JS and EHC, *p* < 0.005), while girls were better than boys on the BB test (*p* < 0.005). Performance scores increased across age groups on all tests (*p* < 0.001). Only for the BB test score, a plateau effect was noted around the age of 12 years. Children and adolescents participating in sports generally outperformed their peers who were not involved in organised sports, on the present KTK3+ test battery. For the second objective, raw score normative values are provided separately for both sexes between 6- to 19-year-olds. In combination with the one-factor structure confirmation, these sex, age, and sport participation effects demonstrate the validity of the test battery. The provided normative values are useful to evaluate MC in children and adolescents from 6 to 19 years old. The use of only four test items that are identical across all ages makes the KTK3+ test battery a practical instrument to assess and compare MC development.

## Introduction

Motor competence (MC) can be defined as the degree of proficient performance in various motor skills as well as the underlying mechanisms such as motor control and coordination (Utesch and Bardid, 2019). The importance of MC lies in its beneficial effect on children's and adolescents' general health and well-being (Stodden et al., 2008; Robinson et al., 2015; Cattuzzo et al., 2016), which is well-documented in literature (Robinson et al., 2015). Across childhood and adolescence, MC is known to be positively associated with many health-related outcomes, including physical activity (Logan et al., 2015), physical fitness (Cattuzzo et al., 2016; Utesch et al., 2019), well-being (Skinner and Piek, 2001), and cognitive health (Haapala, 2013). An inverse relationship between MC and weight status has also been consistently reported (D'Hondt et al., 2013; Cattuzzo et al., 2016). MC involves the mastery of fundamental motor skills, which are the foundation for more advanced and sport specific motor skills (Clark and Metcalfe, 2002; Malina et al., 2004). The construct of MC is measured and assessed in a wide variety of settings and populations, ranging from clinical samples, typically developing children and adolescents, up to (young) elite athletes.

To date, multiple motor test batteries have been developed to enable the evaluation and monitoring of MC in distinct periods throughout the lifespan. A widely used standardised normative and product-oriented test battery is the Körperkoordinationstest für Kinder (“KTK4”) (Kiphard and Schilling, 1974). The KTK4 has been developed to assess gross motor coordination, which refers to one's ability to execute a wide range of motor activities involving whole body movement (Fransen et al., 2014). The original KTK4 assessment tool includes four non-sport specific test items, including jumping sideways (JS), moving sideways (MS), balancing backwards (BB), and hopping for height (HH). All of these tests integrate specific motor skills, such as balance and locomotion, but also rely on components of physical fitness and motor coordination. Kiphard and Schilling (1974) first validated the KTK4 in 1974, providing normative values based on the data of 1,228 German children aged 5–15 years with and without motor and/or health-related issues. Reliability was established for the total raw scores, with Cronbach Alpha scores ranging between 0.80 and 0.96 on the four individual test items, and a total Cronbach Alpha of 0.97 for the overall KTK4 score. Content and construct validity have been documented (Kiphard and Schilling, 1974, 2007), showing moderately strong correlations between the KTK4 scores and other standardised assessment tools such as the Bruininks-Oseretsky Test of Motor Proficiency−2nd Edition (Bruininks and Bruininks, 2005; Fransen et al., 2014), the Motoriktest für Vier- bis Sechsjährige Kinder (Zimmer and Volkamer, 1987; Bardid et al., 2016), as well as the Movement Assessment Battery for Children (Fransen et al., 2014). Furthermore, the KTK4 has been shown to be a good instrument to detect the delayed MC development of children with special needs (i.e., children with brain disorder, behaviour disturbances, or speech impairments), as 91% of these children were identified correctly. Of note, the HH test is often omitted from the KTK4 test protocol in more recent studies due to time constraints and/or safety reasons, especially when applied in adolescents (Pratorius and Milani, 2004; Pion et al., 2014; Deprez et al., 2015b; Mostaert et al., 2016; Lovell et al., 2018; Norjali Wazir et al., 2018). The resulting KTK short form (“KTK3”) has also been demonstrated to represent a valid assessment tool of MC *per se*, with a strong overall correlation between MC scores of the three remaining tests (i.e., JS, MS, and BB) (*r* = 0.98) (Novak et al., 2017).

Because this latter test battery is easy and quick to administer and also has excellent psychometric qualities, the KTK3 is presently still used to detect motor impairment in children, to describe the current level and/or progress of MC in typically developing children, as well as to distinguish children with an adequate level from those with a more advanced level of MC. The KTK3 is also considered to be a useful test battery for longitudinal research into motor development, because the MC tasks involved are characterised by virtually no ceiling effect (Kiphard and Schilling, 1974) and each test item is identical from the age of 5 up until 15 years old (D'Hondt et al., 2013).

Both sex- and age-related normative values are needed to observe the gradual improvement in gross motor coordination across childhood and adolescence. In general, when participants perform a MC test battery, it is often observed that girls have better scores on the balance tasks, whereas there are no clear differences on the locomotion tasks between both sexes (Barnett et al., 2016; Rodrigues et al., 2019). However, these results are mostly seen in the younger age groups, and there is no consensus in the older age groups. Current normative values (Vandorpe et al., 2011b) of the KTK3 are limited to the original reference sample of individuals up to 15 years of age, but motor coordination test batteries are often used in older (sporting/athlete) populations (Pion et al., 2014, 2015; Deprez et al., 2015a,b; Fransen et al., 2017; Norjali Wazir et al., 2018, 2019; O'Brien-Smith et al., 2019). More particularly, the KTK3 has also frequently been used for detection and identification of athletic talents (Vandorpe et al., 2011a, 2012; Deprez et al., 2015b; Pion et al., 2015; Mostaert et al., 2016; Fransen et al., 2017; Norjali Wazir et al., 2018). Therefore, it is important for researchers and practitioners to consider the applicability of this test battery (or an adapted version thereof) in individuals older than 15 years of age, and provide them with normative values that have been validated for older age groups too.

Yet another issue with the KTK4 test battery (and its KTK3 short form) is that only the MS test requires a (limited) degree of object control skill. Nonetheless, object control is considered as a fundamental aspect of MC, in addition to locomotor and balance skills (Gallahue and Donnelly, 2003). These three main motor skill domains should be addressed conjointly to evaluate gross MC in a comprehensive manner. Previous studies have shown the importance of object control skills to develop a physically active lifestyle and, more specifically, for sports performance (Butterfield et al., 2012). Therefore, it seems desirable to add an explicit object control task to the existing KTK3 test protocol. Research of Platvoet et al. (2018) examining 6- to 10-year-old children showed that the KTK3, when supplemented with a catching and throwing task assessing eye-hand coordination (EHC), covers the three abovementioned main motor skill domains (i.e., locomotion, balance, and object control). In addition, these studies revealed good test-retest reliability for all subtests: BB 0.80, MS 0.84, JS 0.95, and EHC 0.87 (Faber et al., 2014; Platvoet et al., 2018). The EHC task, used by Platvoet et al. (2018), requires the individual to throw a tennis ball to the wall with one hand and catch the ball with the other hand. It is a simple and objective test to assess one's ball control and anticipatory capacity. The EHC task is easy to administer in various (large) settings, which may be sports related (e.g., ball and racket sports; Faber et al., 2014; Platvoet et al., 2018). Results of Platvoet et al. (2018) showed an increase in the raw score over the different age groups and a higher score for boys compared to girls. Although based on data from a limited age range, these findings clearly indicated that, when combined with an EHC task, the KTK3 test battery is generally able to cover a broad spectrum of gross motor performance skills and could also discriminate between children with different MC levels.

In the context of the preceding arguments and as suggested by Platvoet et al. (2018), there is a need to expand the current set of norms of the KTK3 and EHC for children and adolescents. This test battery will from now on be referred to as the KTK3+. Therefore, our aim is to validate the combined KTK3+ test battery and provide reference values for both children and adolescents up to emerging adulthood. First, a factor analysis will be performed with the hypothesis that all four test items included in the KTK3+ test battery relate to a single, latent variable: “MC”. Subsequently, the construct validity of the KTK3+ will be investigated by comparing sex, age groups, and participants that are involved or not involved in organised sports participation. Finally, normative values for the KTK3+ test battery will be presented for boys and girls separately as well as per age, between the ages of 6 and 19 years. It is expected that girls will have higher scores on BB, but boys will outperform girls on the EHC task. For JS and MS, we expect no differences in the performance according to sex. Furthermore, we hypothesise that older children and adolescents will systematically outperform their younger counterparts year after year, and that the KTK3+ scores will be higher in those participants who are involved in organised sports.

## Methods

### Participants

Participants in this large-sampled study were children and adolescents aged between 6 and 19 years, who were all based in Flanders (i.e., the Dutch-speaking part of Belgium). Participants were recruited through convenience sampling, since we aspired to test approximately 100 participants per age group, with the expectation of a relatively equal distribution of sex. This sampling method resulted in seven elementary schools, seven secondary schools (with a mix of students in general, technical, and vocational education), two summer camp organisations, and one university. This approach resulted in a total sample of 2,271 participants (1,112 boys and 1,159 girls). Of this sample, 1,248 participants (580 boys and 668 girls) were included in the first part of the study (i.e., the validation process), given that these participants completed a demographic questionnaire in addition to performing the four tests of the KTK3+ test battery. A written informed consent was obtained from each participant. Since the majority of the participants were minors, their parent(s) or their legal representative gave permission for participation in this study. All data were analysed confidentially. This project has been conducted in accordance with the code of Ethics of the World Medical Association (Declaration of Helsinki, 1964, and Declaration of Tokyo, 1975, as revised in 1983) and was approved by the local ethics committee of Ghent University Hospital.

### Procedure

Data collection for this cross-sectional study took place between September 2018 and September 2019. For the construct validation of the KTK3+ test battery, participants were grouped as being involved or not involved in organised sports. To this end, the participants' parent(s) or legal representative were asked to fill out a short demographic survey, also including a binary question (i.e., yes/no) of the involvement in organised sports activity of the child during the school year at the time of measurement. The university students (over 18 years old) filled out this survey themselves.

The testing took place in group in the gymnasium of the participants' school/summer camp/university, during which they were asked to be dressed in light sports clothing and to perform the test battery barefooted. After taking the anthropometric measurements, participants were divided equally among the four MC test items to start the administration of the KTK3+ test battery. When a test item was completed, the participant moved on to a next test item, without using a fixed order. Administration of the anthropometric measurements together with the KTK3+ test battery took approximately 45 min for one group (i.e., consisting of ±25 participants).

For each test day, at least one of the three first authors of this study was present to supervise a group of experienced examiners, who conducted the assessments using standardised instructions in accordance with the original testing manual guidelines (Kiphard and Schilling, 2007; Faber et al., 2014). Before actually conducting each test item of the KTK3+ test battery, participants were given a familiarisation trial for each motor task. Participants were also asked to perform each test item at their best. Test leaders were only allowed to give motivational feedback during actual task performance. However, if they noticed that the participants' test performance was not in line with the prescribed test instructions, they were asked to stop the test, give correctional feedback, and let the participant repeat the test at hand.

### Materials

#### Anthropometry

The anthropometric measurements were conducted with standardised protocols and high-quality mobile equipment. Height was measured with a portable stadiometer with an accuracy of 0.1 cm (Harpenden, Holtain Ltd., Crymych, United Kingdom). Body weight was determined with a bio-electrical impedance scale with an accuracy of 0.1 kg (TANITA BC-420 SMA, Weda B/V., Naarden Holland). Based on these measurements, participants' body mass index (BMI, kg/m^2^) was calculated, and using the most recent international cut-offs suggested by the International Obesity Task Force (IOTF) (Cole and Lobstein, 2012), children and adolescents were classified as being under-weight, normal-weight, overweight, or obese.

### KTK3+ Test Battery

To evaluate children's and adolescents' MC, the use of the KTK3 (Kiphard and Schilling, 1974; Novak et al., 2017) was supplemented with a catching and throwing task (Platvoet et al., 2018) assessing EHC.

#### KTK3

General gross motor coordination was assessed using the KTK3 (Kiphard and Schilling, 1974, 2007; Novak et al., 2017). This is a highly validated, reliable, and product-oriented (quantitative) test instrument that is frequently used on a global scale (Novak et al., 2017). The KTK3 consists of three test items. The first test is jumping sideways (JS), where participants had to jump with two feet over a wooden slat for 15 s. The final score results from the sum of the number of jumps on both trials being provided. For the second test, participants had to move sideways (MS) on a straight line handling two wooden platforms for 20 s. The total score results from summing the number of times participants putted down a wooden platform as well as the number of times participants stepped on the displaced wooden platform during both trials being provided. The third and final test of the KTK3 is balancing backwards (BB) with three trials per balance beam, that is decreasing in width as the test progressed (6.0 cm to 4.5 cm to 3.0 cm). The total amount of steps were counted, with a maximum number of 72 steps (or 8 steps on each trial per balance beam) in total.

#### Eye-Hand Coordination

The EHC test is a valid and reliable product-oriented test (Platvoet et al., 2018) that determines the level of controlling a tennis ball while conducting repetitive movements (i.e., left hand throw, right hand catch, followed by right hand throw, and left hand catch, etc.) as frequently as possible in a time-constrained task of 30 s (Faber et al., 2014). The participants were free to use overhand and/or underhand techniques or a combination of both for throwing and catching. To this end, participants had to stand 1 m from a wall and throw the tennis ball at eye-level in a square (1 m^2^) taped on the wall with the bottom side of the square 1 m above the ground. Participants conducted this test twice, with the number of successful ball catches across both trials resulting in the test score.

### Data Analysis

All data were analysed using SPSS version 26. To test the hypotheses concerning the structure and construct validity of the KTK3+ test battery, the COnsensus-based Standards for the selection of health Measurement INstruments (COSMIN) (Prinsen et al., 2018) was applied. To examine the data for the possible impact of multicollinearity, the Variation Inflation Factor (VIF) was checked. The VIF was calculated three times, each time with the EHC task in relation with one of the KTK3 test items. If the VIF score has a value ranging between 1 and 10, there is no impact of multicollinearity, which implies that the EHC test can be used in combination with the KTK3, resulting in the so-called KTK3+ test battery. Second, multidimensional scaling (MDS) (Borg et al., 2013) was used to conduct a factor analysis in order to verify that all four tests included in the KTK3+ test battery relate to a single, latent variable: “MC”. MDS gives a more detailed insight in the relationship between the four test items (JS, MS, BB, and EHC) by means of a graphical representation. On the accompanying visualisation, the x-axis represents the geometrical distance between the test items, which is to be interpreted as the (dis)similarities between them. Small distances indicate a high correlation or small dissimilarity, whereas large distances indicate a low correlation or large dissimilarity between test items. In this study, a PROXSCAL, non-metrical, MDS with Euclidean distance was applied (Giguère, 2006). The Euclidean distance between the standardised items is a measure of dissimilarity, and its interpretation is in correspondence with the Pearson's correlation analysis. Furthermore, a three-way MANOVA, with sex, age group, and organised sport participation as between-subjects factors, was used to examine differences in KTK3+ test scores. To answer this question in view of the construct validity, only participants with no missing values for organised sport participation and each of the four KTK3+ test items were included in the analyses. For the feasibility of the latter statistical model, age-related differences were tested based on seven distinct age groups (i.e., 6–7.99, 8–9.99, 10–11.99, 12–13.99, 14–15.99, 16–17.99, and 18–19.99 years). In addition, the effect of organised sport participation was inspected, based on two different levels (i.e., participating in organised sports vs. not being involved at all in this kind of activity). Significant interaction and main effects were further examined with Bonferroni *post-hoc* tests. Values of *p* ≤ 0.05 were considered statistically significant for all analyses.

For the second research question, normative values were provided. These values were based on the descriptive statistics of all participants (i.e., also including participants with missing data on the KTK3+ test items), providing raw score normative values per sex and age in years (mean ± SD). In addition to the raw scores collected in our present reference sample, we also wanted to provide standardised values with conversion tables. Therefore, motor quotient (MQ) scores were computed for both boys and girls separately, for each single test item as well as for the total KTK3+ MQ-score. Therefore, individual means and standard deviations were calculated for each sex, age, and test item in order to be able to apply the following formula:

$$\begin{array}{l} {z-score}_{test}=\frac{\left({raw\;score}_{test}-mean_{test}\right)}{{standard\;deviation}_{test}} \end{array}$$

MQ scores could then be derived from the z-scores, again for each single test item and for the total KTK3+ test score, with the following formula, after the example of Pion (2015):

$$\begin{array}{l} MQ_{test}=100+\left({z-score}_{test}\times 15\right) \end{array}$$

For the total KTK3+ MQ-score, a classification on 5 levels of MC based on the normal distribution can be made (Vandorpe et al., 2011b). Values below 70 are seen as an indicative of “severe gross MC disorder,” values between 71 and 85 are considered to represent “moderate gross MC disorder,” values between 86 and 115 are seen as “normal gross MC proficiency,” and MQ-scores between 116 and 130 as “good gross MC proficiency,” whilst values above 131 point to “high gross MC proficiency.”

## Results

First, a detailed overview of the number of participants per sex and age group can be found in Table 1. According to the most recent IOTF cut-off points for BMI (Cole and Lobstein, 2012), 12.9% of the participants in this study could be categorised as being under-weight, 77.2% as normal-weight, 8% as overweight, and 1.9% as obese. This is in agreement with the Flemish prevalence numbers of BMI (Vancoppenolle et al., 2020), which speaks for the representativeness of this sample. The demographic survey revealed that 840 participants (419 boys, 421 girls) were involved in organised sports on a weekly basis during the school year at the moment of testing (i.e., from 1 up to 21 h per week), whereas the remaining 403 participants (161 boys, 242 girls) were not involved in any organised sport activities at the moment of testing, apart from the regular physical education classes at school. A detailed overview of this additional sports related information collected in the subsample can be found per sex and age group in Supplementary Table A.

**Table 1:** Mean and standard deviation (SD) for the anthropometric variables of the participants within the various age groups.

**Age (years)**		**Girls**	***SD***	**Boys**	***SD***	**Total**	***SD***
**6**	***N***	**51**	**73**	**124**			
	Height (cm)	117.66	5.7	118.45	4.59	118.13	5.07
	Weight (kg)	21.03	2.95	21.85	5.42	21.51	4.57
	BMI (kg/m^2^)	15.13	1.13	15.51	3.54	15.35	2.81
**7**	***N***	**51**	**73**	**124**			
	Height (cm)	126.41	5.85	126.74	5.69	126.6	5.73
	Weight (kg)	24.73	3.96	24.43	3.42	24.55	3.64
	BMI (kg/m^2^)	15.41	1.74	15.15	1.27	15.26	1.48
**8**	***N***	**45**	**61**	**106**			
	Height (cm)	131.41	5.65	134	8.61	132.91	7.58
	Weight (kg)	26.5	4.36	28.38	7.43	27.58	6.35
	BMI (kg/m^2^)	15.28	1.76	15.61	1.75	15.47	1.76
**9**	***N***	**63**	**85**	**148**			
	Height (cm)	138.77	7.16	138.25	6.16	138.47	6.58
	Weight (kg)	33.36	8.86	31.12	5.55	32.08	7.21
	BMI (kg/m^2^)	17.14	3.25	16.22	2.21	16.61	2.73
**10**	***N***	**102**	**113**	**215**			
	Height (cm)	144.06	7.69	143.83	6.93	143.94	7.28
	Weight (kg)	36.84	8.53	36.12	8.14	36.46	8.32
	BMI (kg/m^2^)	17.62	3.03	17.33	2.94	17.47	2.98
**11**	***N***	**101**	**107**	**208**			
	Height (cm)	150.81	7.12	148.75	7.83	149.75	7.55
	Weight (kg)	40.14	7.19	38.54	8.36	39.32	7.84
	BMI (kg/m^2^)	17.59	2.61	17.37	3.26	17.48	2.95
**12**	***N***	**152**	**68**	**220**			
	Height (cm)	155.64	7.84	155.13	8.62	155.48	8.07
	Weight (kg)	44.55	8.88	43.67	9.51	44.28	9.07
	BMI (kg/m^2^)	18.35	3.58	18.01	2.83	18.25	3.36
**13**	***N***	**51**	**49**	**100**			
	Height (cm)	163.47	6.02	162.45	9.02	162.97	7.62
	Weight (kg)	51.27	9.3	49.94	12.23	50.62	10.8
	BMI (kg/m^2^)	19.13	2.97	18.72	3.24	18.93	3.09
**14**	***N***	**104**	**111**	**215**			
	Height (cm)	164.01	6.83	168.89	8.49	166.53	8.09
	Weight (kg)	54.43	8.6	55.32	10.97	54.88	9.88
	BMI (kg/m^2^)	20.2	2.76	19.26	2.71	19.71	2.77
**15**	***N***	**82**	**112**	**194**			
	Height (cm)	163.75	6.67	173.82	6.67	169.57	8.31
	Weight (kg)	56.14	10.1	60.45	10.47	58.63	10.51
	BMI (kg/m^2^)	20.89	3.17	19.95	2.91	20.34	3.05
**16**	***N***	**45**	**73**	**118**			
	Height (cm)	165.83	7.41	178.52	7.1	173.68	9.48
	Weight (kg)	57.37	5.99	64.41	8.69	61.73	8.47
	BMI (kg/m^2^)	20.98	2.79	20.16	1.93	20.47	2.32
**17**	***N***	**84**	**64**	**148**			
	Height (cm)	165.99	6.58	179.38	7.03	171.78	9.49
	Weight (kg)	59.86	9	66.92	9.27	62.92	9.74
	BMI (kg/m^2^)	21.75	3.14	20.82	2.79	21.35	3.02
**18**	***N***	**172**	**96**	**268**			
	Height (cm)	167.93	5.75	179.78	6.62	172.17	8.32
	Weight (kg)	60.99	7.5	70.86	10.21	64.53	9.78
	BMI (kg/m^2^)	21.63	2.45	21.88	2.6	21.72	2.5
**19**	***N***	**51**	**22**	**73**			
	Height (cm)	167.42	6.1	183.4	6.44	172.23	9.61
	Weight (kg)	62.1	8.93	75.38	7.46	66.1	10.45
	BMI (kg/m^2^)	22.12	2.66	22.5	2.87	22.23	2.71

**Figure 1:**
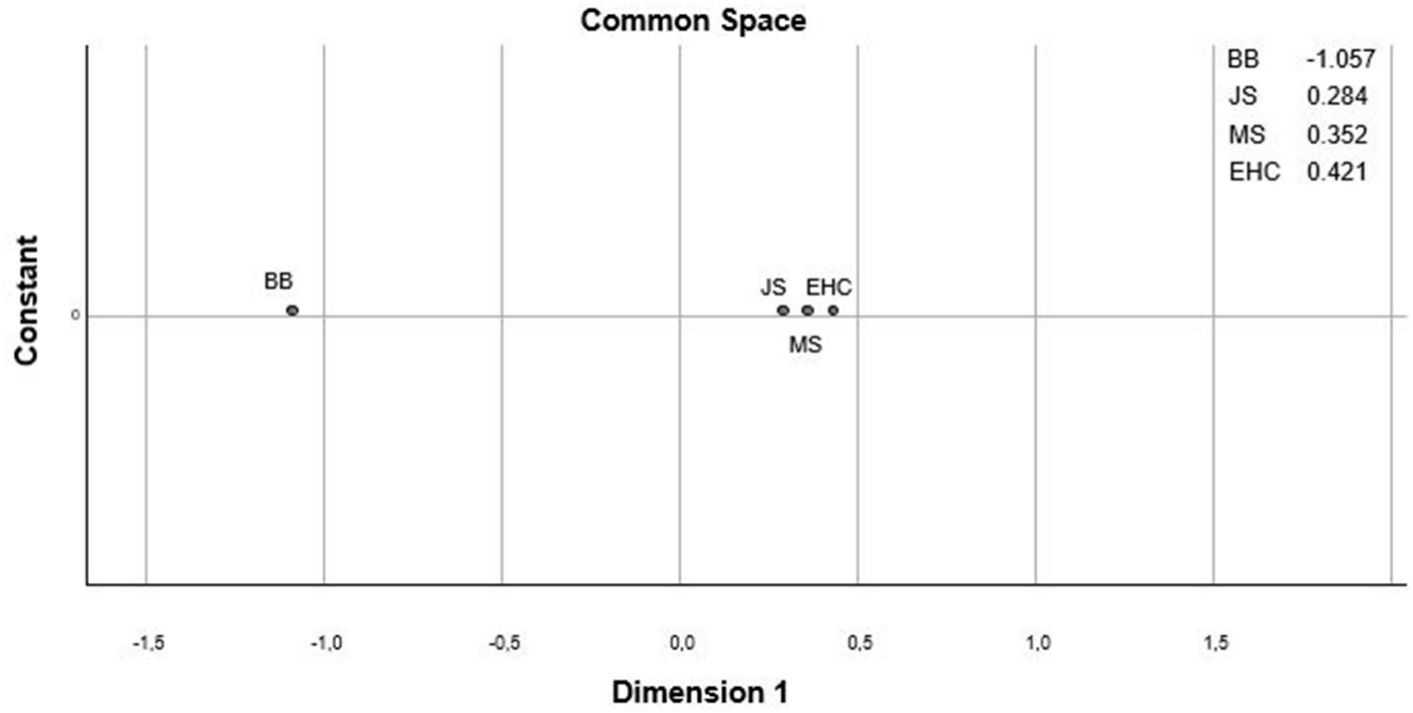
One-dimensional configuration for the four items of the KTK3+ test battery. Dimension 1 represents the geometrical distance between the test items (JS, jumping sideways; MS, moving sideways; BB, balancing backwards; EHC, eye-hand coordination) translated as the (dis)similarities between the items, with small distances indicating a high correlation or small dissimilarity and large distances indicating a low correlation or large dissimilarity.

For the first research question, the factor structure of KTK3+ test battery was examined. The VIF for all KTK-3 test items in relation to the EHC task varied between 1 and 10, indicating that all four tests could remain combined (VIF_JS_ = 2.812; VIF_MS_ = 2.511; VIF_BB_= 1.604). Afterwards, a one- to two-dimensional structure of the four items within the KTK3+ test battery was examined with the non-metric MDS analysis. Different fit indices were used to assess model fit. The scree test as well as the stress and fit measures revealed that a one-dimensional factor structure was most suitable. Both the raw stress score and Tucker's coefficient supported this, with good outcome scores (0.001 and 1.00, respectively) (Lorenzo-Seva and Ten Berge, 2006). These measures show that 99.9% of the distances are explained by the one-dimensional configuration, indicating an excellent structure of the KTK3+ test battery (see Figure 1). The correlations between the four KTK3+ test items (i.e., JS, MS, BB, and EHC) ranged from moderate to very good (*r* = 0.453–0.799) (Schober et al., 2018), reflecting the one-dimensional structure of the KTK3+ test battery (Table 2).

**Table 2:** Correlations between the four items of the KTK3+ test battery in total and per age group.

	**MS**	**BB**	**EHC**
**6–7.99 years old**			
JS	0.347^**^	0.538^**^	0.489^**^
MS		0.342^**^	0.268^**^
BB			0.184^**^
**8–9.99 years old**			
JS	0.301^**^	0.521^**^	0.615^**^
MS		0.354^**^	0.297^**^
BB			0.242^**^
**10–11.99 years old**			
JS	0.375^**^	0.413^**^	0.554^**^
MS		0.466^**^	0.233^**^
BB			0.163^*^
**12–13.99 years old**			
JS	0.564^**^	0.450^**^	0.348^**^
MS		0.438^**^	0.182^**^
BB			0.144^*^
**14–15.99 years old**			
JS	0.543^**^	0.433^**^	0.463^**^
MS		0.392^**^	0.293^**^
BB			0.216^**^
**16–17.99 years old**			
JS	0.569^**^	0.388^**^	0.504^**^
MS		0.291^**^	0.367^**^
BB			0.254^**^
**18–19.99 years old**			
JS	0.516^**^	0.372^**^	0.251^**^
MS		0.341^**^	0.178^**^
BB			0.067
**Total**			
JS	0.752^**^	0.584^**^	0.799^**^
MS		0.533^**^	0.695^**^
BB			0.453^**^

Correlation is significant at the 0.05 level (^*^) or 0.01 level (^**^).

The construct validity of the KTK3+ test battery was examined using a three-way MANOVA to compare differences in test scores (i.e., JS, MS, BB, and EHC) according to sex, age group, and organised sport participation.

There was neither a significant three-way multivariate interaction effect, nor an interaction effect for sex^*^organised sport participation and age group^*^organised sport participation at the multivariate level. A significant multivariate interaction effect was found, however, between sex and age group [*F*_(24, 4860)_ = 2.089 and *p* = 0.001], as well as a significant main effect for sex [*F*_(4, 1211)_ = 39.508; *p* < 0.001], age group [*F*_(24, 4860)_ = 58.489; *p* < 0.001], and organised sport participation [*F*_(4, 1211)_ = 15.040; *p* < 0.001].

**Figure 2:**
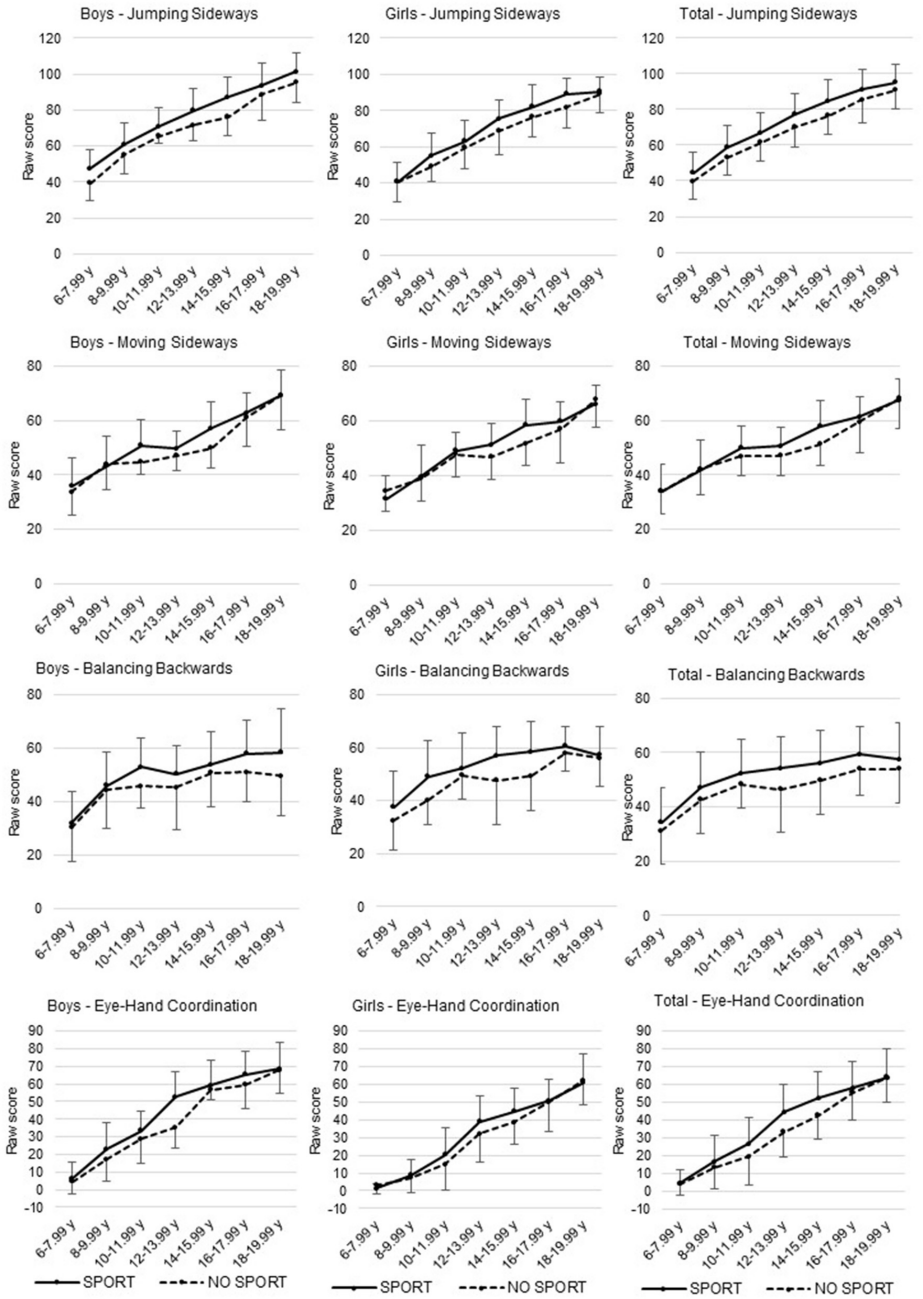
Differences in raw scores on each KTK3+ test item for the total sample and between sex, age groups, and organised sport participation.

Univariate sex^*^age group interaction effects tended to be significant for the JS test and reached significance for the EHC task (*p* = 0.063 and *p* = 0.002; respectively). A closer inspection of this interaction showed that boys demonstrated better scores on the JS test and EHC task in each age group compared to girls. However, for the JS test, the difference between boys and girls was greater in older (≥16.00 years old) than in younger (<16.00 years old) age groups. For the EHC test, in contrast, the sex difference became smaller with increasing age. Univariate main effect revealed that girls scored better on the BB test (*p* = 0.003) when compared to boys. For the MS test, a tendency toward a main effect of sex was observed (*p* = 0.080) in favour of the boys. Regardless of sex and organised sport participation, a significant increase in test scores was found for each age group on the four test items of the KTK3+ test battery (all *p* < 0.001). *Post-hoc* Bonferroni tests showed differences between all age groups for the JS test, MS test, and EHC task. For the BB test, only a significant difference in scores emerged in the younger age groups (i.e., until 10- to 11.99-year-olds), compared to the older age groups (Table 3). Regardless of sex and age group, it was found that children and adolescents who are involved in organised sports performed significantly better on all the KTK3+ test items when compared to peers who are not involved in any organised sports (all *p* < 0.005). Figure 2 displays the differences for the raw scores on all four test items for the total group and according to sex, age groups, and organised sport participation.

**Table 3:** Means and standard deviations (SD) from the raw scores on each KTK3+ test battery for the age categories, with the *F*, (*df*), *p*, and partial η^2^-values of the MANOVA.

**Age** **(years)**	**6–7.99** **mean ±** **SD**	**8–9.99** **mean ±** **SD**	**10–11.99** **mean ±** **SD**	**12–13.99** **mean ±** **SD**	**14–15.99** **mean ±** **SD**	**16–17.99** **mean ±** **SD**	**18–19.99** **mean ±** **SD**	**Sex**	**Age** **Group**	**Sport**	**Age** **Group × Sex**	**Age Group ×** **Sport**	**Sex** **× Sport**	**Age Group** **× Sport × Sex**
**Multivariate**								*F* = 39.508	*F* = 58.489	*F* = 15.040	*F* = 2.089	*F* = 1.314	*F* = 1.471	*F* = 1.363
								(4; 1,211)	(24; 4,860)	(4; 1,211)	(24; 4,860)	(24; 4,860)	(4; 1,211)	(24; 4,860)
								*p* < 0.001	*p* < 0.001	*p* < 0.001	*p* = 0.001	*p* = 0.140	*p* = 0.209	*p* = 0.111
								η^2^ = 0.115	η^2^ = 0.224	η^2^ = 0.047	η^2^ = 0.010	η^2^ = 0.006	η^2^ = 0.005	η^2^ = 0.007
**JS**	43 ± 11	58 ± 12	66 ± 12	75 ± 12	83 ± 12	90 ± 12	93 ± 11	*F* = 33.991	*F* = 468.884	*F* = 49.541	*F* = 1.998	*F* = 1.023	*F* = 3.415	*F* = 0.878
	^a^	^b^	^c^	^d^	^e^	^f^	^f, g^	(1)	(6)	(1)	(6)	(6)	(1)	(6)
								*p* < 0.001	*p* < 0.001	*p* < 0.001	*p* = 0.063	*p* = 0.409	*p* = 0.065	*p* = 0.510
								η^2^ = 0.027	η^2^ = 0.698	η^2^ = 0.039	η^2^ = 0.010	η^2^ = 0.005	η^2^ = 0.003	η^2^ = 0.004
**MS**	34 ± 9	42 ± 11	49 ± 8	50 ± 7	57 ± 10	61 ± 9	67 ± 10	*F* = 3.075	*F* = 275.823	*F* = 8.521	*F* = 1.363	*F* = 2.309	*F* = 0.478	*F* = 0.632
	^a^	^b^	^c^	^c, d^	^e^	^f^	^g^	(1)	(6)	(1)	(6)	(6)	(1)	(6)
								*p* = 0.080	*p* < 0.001	*p* = 0.004	*p* = 0.226	*p* = 0.032	*p* = 0.489	*p* = 0.705
								η^2^ = 0.003	η^2^ = 0.577	η^2^ = 0.007	η^2^ = 0.007	η^2^ = 0.011	η^2^ = 0.000	η^2^ = 0.003
**BB**	33 ± 13	46 ± 13	52 ± 12	52 ± 13	55 ± 12	58 ± 10	56 ± 13	*F* = 8.954	*F* = 70.436	*F* = 31.961	*F* = 0.547	*F* = 0.316	*F* = 0.036	*F* = 2.052
	^a^	^b^	^b, d, e, g^	^c, d, e, g^	^c, d, e, f, g^	^e, f, g^	^c, d, e, f, g^	(1)	(6)	(1)	(6)	(6)	(1)	(6)
								*p* = 0.003	*p* < 0.001	*p* < 0.001	*p* = 0.772	*p* = 0.929	*p* = 0.849	*p* = 0.056
								η^2^ = 0.007	η^2^ = 0.258	η^2^ = 0.026	η^2^ = 0.003	η^2^ = 0.002	η^2^ = 0.000	η^2^ = 0.010
**EHC**	4 ± 7	16 ± 13	25 ± 15	42 ± 16	51 ± 15	57 ± 15	64 ± 15	*F* = 105.857	*F* = 501.803	*F* = 16.594	*F* = 3.438	*F* = 2.878	*F* = 2.447	*F* = 0.619
	^a^	^b^	^c^	^d^	^e^	^f^	^g^	(1)	(6)	(1)	(6)	(6)	(1)	(6)
								*p* < 0.001	*p* < 0.001	*p* < 0.001	*p* = 0.002	*p* = 0.009	*p* = 0.118	*p* = 0.715
								η^2^ = 0.080	η^2^ = 0.712	η^2^ = 0.013	η^2^ = 0.017	η^2^ = 0.014	η^2^ = 0.002	η^2^ = 0.003

*JS, jumping sideways; MS, moving sideways; BB, balancing backwards; EHC, eye-hand coordination*.

a, b, c, d, e, f, g *A mean is significantly different from another mean if they have other superscript letters*.

For the second objective, normative values were established from the collected KTK3+ data in boys and girls aged between 6 and 19 years. Based on the raw scores of the four test items (i.e., JS, MS, BB, and EHC), percentile values at P5, P25, P50, P75, and P90 according to sex and age (per year) were calculated and presented (Table 4). Raw test scores were also converted into standardised values, based on the abovementioned z- and MQ-score formulas. The conversion tables from raw test scores into standardised values can be found in the Supplementary Material for each sex, age (per year), and KTK3+ test item separately as well as a conversion table to determine the total MQ-score based on the actual gross motor skill performances (see addendum: Supplementary Tables B–E).

**Table 4:** Overview of the raw scores that correspond with the 5th, 25th, 50th, 75th, and 95th percentile on each of the four test items of the KTK3+ test battery (JS, jumping sideways; MS, moving sideways; BB, balancing backwards; EHC, eye-hand coordination) according to sex and age (per year).

**Age (years)**	**6**					**7**					**8**					**9**					**10**					**11**					**12**					**13**					**14**					**15**					**16**					**17**					**18**					**19**				
	**P5**	**P25**	**P50**	**P75**	**P95**	**P5**	**P25**	**P50**	**P75**	**P95**	**P5**	**P25**	**P50**	**P75**	**P95**	**P5**	**P25**	**P50**	**P75**	**P95**	**P5**	**P25**	**P50**	**P75**	**P95**	**P5**	**P25**	**P50**	**P75**	**P95**	**P5**	**P25**	**P50**	**P75**	**P95**	**P5**	**P25**	**P50**	**P75**	**P95**	**P5**	**P25**	**P50**	**P75**	**P95**	**P5**	**P25**	**P50**	**P75**	**P95**	**P5**	**P25**	**P50**	**P75**	**P95**	**P5**	**P25**	**P50**	**P75**	**P95**	**P5**	**P25**	**P50**	**P75**	**P95**	**P5**	**P25**	**P50**	**P75**	**P95**
**JS**																																																																										
Boys	26	35	40	45	64	33	43	50	57	66	40	50	56	63	74	39	59	66	72	84	48	61	68	75	84	53	67	73	83	94	59	70	78	86	103	61	74	80	91	101	63	75	84	90	101	62	77	85	94	106	64	80	89	100	113	67	86	94	102	115	82	91	99	107	116	69	81	94	105	122
Girls	20	28	37	44	58	27	36	45	52	61	28	45	56	62	71	38	51	59	66	82	41	57	64	72	89	50	61	70	76	86	57	66	74	82	96	50	67	74	86	99	57	70	77	84	97	58	71	79	88	101	67	78	85	92	104	65	78	85	90	99	75	84	90	95	106	71	83	88	95	107
Total	23	32	39	45	59	31	40	48	55	63	33	47	56	63	72	38	54	62	70	83	46	59	67	74	84	51	64	71	79	91	58	68	76	83	96	56	70	79	88	100	59	72	80	88	99	61	74	83	91	104	67	78	87	97	109	66	81	89	95	109	77	86	92	99	112	70	83	89	99	110
**MS**																																																																										
Boys	22	31	36	40	47	15	26	38	44	52	19	39	43	49	54	22	42	47	51	58	37	44	48	54	59	29	45	50	56	65	39	47	52	59	68	40	49	55	58	64	33	49	55	63	72	38	51	56	64	74	44	54	62	68	79	51	57	62	72	80	52	64	71	76	87	51	59	66	73	90
Girls	23	29	33	36	43	15	21	36	40	48	21	38	43	47	54	20	36	44	50	56	33	44	50	54	63	36	46	51	56	63	38	47	51	57	65	31	48	54	60	69	40	49	55	62	71	38	52	58	65	75	45	52	58	64	78	44	54	60	65	75	54	62	67	71	81	55	63	67	72	87
Total	24	30	34	39	45	15	24	36	42	51	20	39	43	48	54	21	40	46	50	57	36	44	49	54	60	35	45	50	56	64	39	47	51	58	65	38	48	54	60	69	39	49	55	62	71	38	52	57	64	74	44	53	60	67	77	46	55	61	68	77	52	63	68	73	83	54	61	67	73	87
**BB**																																																																										
Boys	9	19	27	37	47	17	30	36	44	54	20	34	45	52	60	25	34	46	57	66	26	39	47	56	67	26	44	54	61	70	26	42	52	60	69	28	41	53	62	70	31	42	55	62	71	24	40	51	60	69	29	49	57	66	72	30	43	51	60	72	28	45	54	66	72	15	37	50	62	72
Girls	8	23	33	41	52	20	28	38	48	64	19	39	48	58	66	21	39	47	60	72	20	44	52	59	67	30	49	55	63	72	32	48	56	65	72	29	43	55	65	72	30	44	55	63	72	36	49	58	65	72	43	53	60	65	72	38	49	58	66	72	30	50	57	67	72	41	47	53	62	72
Total	8	20	30	38	50	18	29	37	46	55	20	36	46	53	64	24	38	47	59	68	24	41	49	58	66	27	46	55	62	71	30	46	55	63	72	29	42	54	63	72	31	44	55	62	72	29	45	53	61	71	34	51	59	65	72	36	46	56	63	72	29	49	57	66	72	33	46	53	62	72
**EHC**																																																																										
Boys	0	0	1	4	20	0	2	5	13	33	0	6	16	30	43	2	11	28	37	50	6	28	34	45	56	15	29	39	45	52	20	41	45	55	73	20	43	51	57	78	29	44	55	62	73	33	47	56	65	80	41	52	63	72	88	33	51	62	69	89	43	58	68	78	93	42	59	67	75	91
Girls	0	0	0	1	4	0	0	1	4	16	0	1	4	8	29	1	4	11	23	33	2	5	14	26	43	0	10	21	36	52	7	19	30	41	52	17	29	39	53	62	17	31	41	50	65	19	34	42	52	62	25	38	50	57	65	24	38	47	54	64	35	51	59	69	88	43	54	62	76	92
Total	0	0	0	2	14	0	0	3	8	28	0	3	9	23	41	1	6	20	32	45	2	9	25	37	50	3	19	34	44	52	10	22	36	45	59	17	31	49	54	76	20	37	48	58	69	25	39	51	61	76	30	45	56	67	87	27	44	52	62	83	38	53	62	73	89	42	56	63	76	91

*JS, jumping sideways; MS, moving sideways; BB, balancing backwards; EHC, eye-hand coordination*.

## Discussion

The present study evaluated the validity of the combined KTK3+ test battery for evaluating MC in children and adolescents up to emerging adulthood. First, this study showed an excellent structure validity. Second, our results revealed that the KTK3+ test battery was able to differentiate between gross motor coordination performances according to sex, age groups, and organised sport participation. Next to the validation process of this particular assessment tool, normative values were provided for children and adolescents (i.e., for boys and girls separately, aged 6–19 years).

The structure validity of the KTK3+ test battery was checked using MDS. By adding the EHC-task (Platvoet et al., 2018) to the KTK3 (Novak et al., 2017), the three motor skill domains (i.e., locomotor skills, balance skills, and object control skills) are all addressed in one comprehensive, quick, and easy to administer test battery. Although the four test items each assess a slightly different skill domain of gross MC, they indeed all relate to the same, single construct. This outcome was previously found by Platvoet et al. (2018), who used both the KTK3 and EHC-task in 6- to 10-year-old primary school children. The present validation study showed that the combined KTK3+ test battery can also be used to evaluate gross MC in a wider age range. Furthermore, normative values were provided for sex, age (per year), and each test item separately. However, the normative values provided in the present study are based on raw performance scores. Therefore, conversion tables with standardised values (i.e., MQ scores for each test item as well as the test battery in total) were added as Supplementary Material Table (B–E). In this way, the KTK3+ test battery can be used by practitioners and researchers to make a global evaluation of the level of MC of the target group. Nonetheless, the MQ-scores are not discussed further in this article, because this is beyond the scope of the predetermined research questions.

In agreement with previous research, sex differences emerged on all tests during childhood and (early) adolescence (Iivonen and Sääkslahti, 2014; Barnett et al., 2016; Rodrigues et al., 2019). Our study revealed that boys systematically outperformed girls on three out of the four KTK3+ test items, while girls outperformed boys on the BB test. These findings are consistent with the assumption that sex appears to relate differently to various aspects of gross MC and can be explained by biological influences on motor development (Barnett et al., 2016). Boys had significantly higher scores than girls on JS, and this difference was even more pronounced among the older age groups. This finding could also be explained by an underlying physical and physiological factor, as Vandorpe et al. (2011b) already suggested that strength and/or endurance are underlying requirements for a good performance on the JS. When it comes to MS, the mean scores for the boys were also found to be higher than in girls, but this only tended to be a significant effect. This borderline significant finding is in accordance with previous literature, where mixed results are seen when it comes to sex differences in locomotion (Barnett et al., 2010). Due to these sex differences, normative values of the KTK3+ test battery were provided separately for boys and girls at all ages in the present paper, since the differences between sex remained over time or with increasing age, even after puberty.

The significant improvement in MC with age in the current study, where participants in older age groups scored significantly better than their counterparts in the younger age group(s), is in accordance with the studies of Ahnert et al. (2010) and Vandorpe et al. (2011b). Our results are also in line with the findings in the study of Rodrigues et al. (2019) and the systematic review of Barnett et al. (2016), showing a positive relation between age and MC during and after adolescence. However, in our study the increase in motor performance with age was less pronounced after puberty. Therefore, separate age-related normative values seem warranted for children and adolescents as presented in our Supplementary Material Table (B–E). It should be noted that a floor effect was observed in the EHC task among the 6- to 7-year-olds. Since a complex spatial-temporal relationship between our visual system and manual motor system is needed to complete the EHC task, it might be that the EHC test is rather challenging for these specific young(er) age groups, which was also observed by Rizzo et al. (2017) and Platvoet et al. (2018). Another possible reason could be the smaller anthropometric measurements of younger children (i.e., hand size and arm length), also complicating ball catching and throwing. The current study only found a plateau effect on the BB task, starting from the age of 12 years, which could indicate that dynamic balance approaches the mature performance level around that age (Largo et al., 2001). However, for both the EHC and BB, test variance was still seen between the percentile scales, which is also observable in Table 4.

Since the goal of this paper was to provide normative values validated for older age groups in both sporting and non-sporting populations, the KTK3+ test battery proved to be a highly practical and valuable tool. With this test battery, differences in performance between both groups were examined. Participants who were not involved in any organised sport activities scored systematically lower on all motor tasks included in the KTK3+ test battery when compared to peers who were involved in organised sports on a weekly basis. This difference was seen both in the younger and older age groups; however, the effect size found in this study was relatively low. Previous research has already shown that physical activity, including organised sport participation, has a positive impact on the development of MC in childhood (Robinson et al., 2015). Additionally, taking into account studies in sports settings that already made use of the KTK3 (Vandorpe et al., 2012; Sögüt, 2017), the combined KTK3+ test battery could even be utilised in talent identification and development environments to detect the high MC proficient movers, as suggested in the systematic review of O'Brien-Smith et al. (2019).

However, some limitations need to be addressed. First, researchers should be aware that the normative values presented in this study are solely based on a reference sample of Flemish children. Therefore, caution is needed when using these standardised values (MQ-scores) when assessing children elsewhere, given the effect of context and culture on motor development. Future studies should extend upon this, and add normative data from other countries to better understand motor competence on a global scale (i.e., its factorial structure and measurement invariance across groups). Second, this study has a cross-sectional design. Longitudinal and experimental studies should be conducted to gain more insight in the development of motor competence throughout the lifespan. Third, the participation in organised sports was only surveyed in a binary way. Therefore, future research would benefit from also including measurements of type of sport participation, training history, training intensity, as well as other forms of PA, such as participation in unorganised sports, active transport, and school-based PA. In addition, exploring psychosocial factors such as socioeconomic status and parental support as well as enjoyment could be helpful. Lastly, a floor effect in the EHC task and a plateau effect in the BB task were observed. This is relevant information for researchers using these test protocols within children and adolescent populations.

The present study revealed that the KTK3+ test battery is a valuable and valid tool for assessing MC as a single construct in children and adolescents from 6- to 19-year-olds. The test battery is able to provide normative values that are sex and age specific and to discriminate gross motor performances between consecutive sex, age groups, and organised sport participation. It creates opportunities for practitioners to better meet children's and adolescents' individual developmental MC needs and to evaluate the effectiveness of their own practices. The fact that the KTK3+ test battery can be used in children and adolescents from 6- to 19-year-olds makes it a tool of high practical value, especially for the longitudinal follow-up of MC. The large age range, easy test protocol, and quick setup are major strengths of the KTK3+ test battery. In addition, the KTK3+ test battery measures the whole range of gross motor domains, including aspects of balance skills, locomotion, and object control skills.

## Data Availability Statement

The data presented in this study is available at: https://osf.io/xcvsp/?view_only=0a86a41f107043c2ae26ff07258d818f.

## Ethics Statement

The studies involving human participants were reviewed and approved by Ghent University Hospital. Written informed consent to participate in this study was provided by the participants' legal guardian/next of kin.

## Author Contributions

EC, FL, MM, and ML were involved in the conceptualisation of the study. EC, FL, and MM were involved in data collection. EC, FL, MM, ED'H, FD, and ML were involved in the writing of the manuscript. All authors contributed to and approved the final version of the manuscript.

## Conflict of Interest

The authors declare that the research was conducted in the absence of any commercial or financial relationships that could be construed as a potential conflict of interest.
