# Supplementary material for: Validation of a Motor Competence Assessment Tool for Children and Adolescents (KTK3+) With Normative Values for 6- to 19-Year-Olds
Source: Front Physiol. 2021 Jun 23;12:652952. doi: 10.3389/fphys.2021.652952 (PMC8260948; doi:10.3389/fphys.2021.652952)
Supplement: Supplementary file 1 [file Data_Sheet_1.ZIP › Supplementary_Material_Table_A.docx]

| Supplementary Material | | | |  | |  | |  | |  | |  | |  | |  | |  | |  | |  | |  |  | |  |  | |
| --- | --- | --- | --- | --- | --- | --- | --- | --- | --- | --- | --- | --- | --- | --- | --- | --- | --- | --- | --- | --- | --- | --- | --- | --- | --- | --- | --- | --- | --- |
| **Table A.** Numbers of participants who did or did not participate in organised sports activities. | | | | | | | | | | | | | |  | |  | |  | |  | |  | |  |  | |  |  | |
| Age (years) | 6 | | 7 | | | | 8 | | | | 9 | | | | 10 | | | | 11 | | | | 12 | | |  | | |  |
|  | sport | no sport | sport | | no sport | | sport | | no sport | | sport | | no sport | | sport | | no sport | | sport | | no sport | | sport | | no sport |  | | |  |
| Boys | 44 | 20 | 45 | | 15 | | 41 | | 9 | | 26 | | 7 | | 16 | | 1 | | 22 | | 5 | | 20 | | 6 |  | | |  |
| Girls | 33 | 14 | 33 | | 8 | | 23 | | 7 | | 26 | | 3 | | 28 | | 7 | | 16 | | 7 | | 40 | | 10 |  | | |  |
| Total | 77 | 34 | 78 | | 23 | | 64 | | 16 | | 52 | | 10 | | 44 | | 8 | | 38 | | 12 | | 60 | | 16 |  | | |  |
| Age (years) | 13 | | 14 | | | | 15 | | | | 16 | | | | 17 | | | | 18 | | | | 19 | | | Total | | | |
|  | sport | no sport | sport | | no sport | | sport | | no sport | | sport | | no sport | | sport | | no sport | | sport | | no sport | | sport | | no sport | sport | | | no sport |
| Boys | 16 | 7 | 39 | | 3 | | 47 | | 5 | | 30 | | 18 | | 19 | | 5 | | 46 | | 46 | | 8 | | 14 | 419 | | | 161 |
| Girls | 15 | 7 | 43 | | 15 | | 38 | | 11 | | 24 | | 7 | | 24 | | 10 | | 65 | | 100 | | 13 | | 36 | 421 | | | 242 |
| Total | 31 | 14 | 82 | | 18 | | 85 | | 16 | | 54 | | 25 | | 43 | | 15 | | 111 | | 146 | | 21 | | 50 | 840 | | | 403 |
