# Supplementary material for: Validation of a Motor Competence Assessment Tool for Children and Adolescents (KTK3+) With Normative Values for 6- to 19-Year-Olds
Source: Front Physiol. 2021 Jun 23;12:652952. doi: 10.3389/fphys.2021.652952 (PMC8260948; doi:10.3389/fphys.2021.652952)
Supplement: Supplementary file 1 [file Data_Sheet_1.ZIP › Supplementary_Material_Table_B.docx]

Supplementary Material

Turning tables were made for the four tests of the KTK3+, together with a table to calculate the total MQ-score. The tables are separately for children and adolescents, due to influences of maturation at the older ages.

**Tables B1 to B8** display the raw performance scores for girls and boys on the JS, MS, BB and EHC task of the KTK3+ for children.

| **Table B1.** Raw scores elementary school 'Jumping Sideways' (JS) (Girls) | | | | | | |
| --- | --- | --- | --- | --- | --- | --- |
| **Raw Score / Age (years)** | **6-6.99** | **7-7.99** | **8-8.99** | **9-9.99** | **10-10.99** | **11-11.99** |
| **1** | 52 | 36 | 36 | 27 | 29 | 11 |
| **2** | 53 | 38 | 37 | 28 | 31 | 12 |
| **3** | 54 | 39 | 39 | 30 | 32 | 14 |
| **4** | 56 | 40 | 40 | 31 | 33 | 15 |
| **5** | 57 | 42 | 41 | 32 | 34 | 16 |
| **6** | 58 | 43 | 42 | 33 | 35 | 18 |
| **7** | 60 | 45 | 44 | 35 | 36 | 19 |
| **8** | 61 | 46 | 45 | 36 | 37 | 20 |
| **9** | 62 | 48 | 46 | 37 | 38 | 22 |
| **10** | 63 | 49 | 47 | 38 | 39 | 23 |
| **11** | 65 | 51 | 49 | 40 | 41 | 24 |
| **12** | 66 | 52 | 50 | 41 | 42 | 26 |
| **13** | 67 | 54 | 51 | 42 | 43 | 27 |
| **14** | 69 | 55 | 52 | 44 | 44 | 28 |
| **15** | 70 | 57 | 53 | 45 | 45 | 29 |
| **16** | 71 | 58 | 55 | 46 | 46 | 31 |
| **17** | 73 | 60 | 56 | 47 | 47 | 32 |
| **18** | 74 | 61 | 57 | 49 | 48 | 33 |
| **19** | 75 | 63 | 58 | 50 | 50 | 35 |
| **20** | 77 | 64 | 60 | 51 | 51 | 36 |
| **21** | 78 | 66 | 61 | 52 | 52 | 37 |
| **22** | 79 | 67 | 62 | 54 | 53 | 39 |
| **23** | 81 | 68 | 63 | 55 | 54 | 40 |
| **24** | 82 | 70 | 65 | 56 | 55 | 41 |
| **25** | 83 | 71 | 66 | 57 | 56 | 42 |
| **26** | 85 | 73 | 67 | 59 | 57 | 44 |
| **27** | 86 | 74 | 68 | 60 | 58 | 45 |
| **28** | 87 | 76 | 70 | 61 | 60 | 46 |
| **29** | 88 | 77 | 71 | 63 | 61 | 48 |
| **30** | 90 | 79 | 72 | 64 | 62 | 49 |
| **31** | 91 | 80 | 73 | 65 | 63 | 50 |
| **32** | 92 | 82 | 74 | 66 | 64 | 52 |
| **33** | 94 | 83 | 76 | 68 | 65 | 53 |
| **34** | 95 | 85 | 77 | 69 | 66 | 54 |
| **35** | 96 | 86 | 78 | 70 | 67 | 56 |
| **36** | 98 | 88 | 79 | 71 | 69 | 57 |
| **37** | 99 | 89 | 81 | 73 | 70 | 58 |
| **38** | 100 | 91 | 82 | 74 | 71 | 59 |
| **39** | 102 | 92 | 83 | 75 | 72 | 61 |
| **40** | 103 | 94 | 84 | 76 | 73 | 62 |
| **41** | 104 | 95 | 86 | 78 | 74 | 63 |
| **42** | 106 | 96 | 87 | 79 | 75 | 65 |
| **43** | 107 | 98 | 88 | 80 | 76 | 66 |
| **44** | 108 | 99 | 89 | 82 | 77 | 67 |
| **45** | 110 | 101 | 91 | 83 | 79 | 69 |
| **46** | 111 | 102 | 92 | 84 | 80 | 70 |
| **47** | 112 | 104 | 93 | 85 | 81 | 71 |
| **48** | 113 | 105 | 94 | 87 | 82 | 72 |
| **49** | 115 | 107 | 96 | 88 | 83 | 74 |
| **50** | 116 | 108 | 97 | 89 | 84 | 75 |
| **51** | 117 | 110 | 98 | 90 | 85 | 76 |
| **52** | 119 | 111 | 99 | 92 | 86 | 78 |
| **53** | 120 | 113 | 100 | 93 | 88 | 79 |
| **54** | 121 | 114 | 102 | 94 | 89 | 80 |
| **55** | 123 | 116 | 103 | 95 | 90 | 82 |
| **56** | 124 | 117 | 104 | 97 | 91 | 83 |
| **57** | 125 | 119 | 105 | 98 | 92 | 84 |
| **58** | 127 | 120 | 107 | 99 | 93 | 86 |
| **59** | 128 | 122 | 108 | 101 | 94 | 87 |
| **60** | 129 | 123 | 109 | 102 | 95 | 88 |
| **61** | 131 | 124 | 110 | 103 | 96 | 89 |
| **62** | 132 | 126 | 112 | 104 | 98 | 91 |
| **63** | 133 | 127 | 113 | 106 | 99 | 92 |
| **64** | 135 | 129 | 114 | 107 | 100 | 93 |
| **65** | 136 | 130 | 115 | 108 | 101 | 95 |
| **66** | 137 | 132 | 117 | 109 | 102 | 96 |
| **67** | 138 | 133 | 118 | 111 | 103 | 97 |
| **68** | 140 | 135 | 119 | 112 | 104 | 99 |
| **69** | 141 | 136 | 120 | 113 | 105 | 100 |
| **70** | 142 | 138 | 121 | 114 | 106 | 101 |
| **71** | 144 | 139 | 123 | 116 | 108 | 102 |
| **72** | 145 | 141 | 124 | 117 | 109 | 104 |
| **73** | 146 | 142 | 125 | 118 | 110 | 105 |
| **74** | 148 | 144 | 126 | 119 | 111 | 106 |
| **75** | 149 | 145 | 128 | 121 | 112 | 108 |
| **76** | 150 | 147 | 129 | 122 | 113 | 109 |
| **77** |  | 148 | 130 | 123 | 114 | 110 |
| **78** |  | 150 | 131 | 125 | 115 | 112 |
| **79** |  |  | 133 | 126 | 117 | 113 |
| **80** |  |  | 134 | 127 | 118 | 114 |
| **81** |  |  | 135 | 128 | 119 | 116 |
| **82** |  |  | 136 | 130 | 120 | 117 |
| **83** |  |  | 138 | 131 | 121 | 118 |
| **84** |  |  | 139 | 132 | 122 | 119 |
| **85** |  |  | 140 | 133 | 123 | 121 |
| **86** |  |  | 141 | 135 | 124 | 122 |
| **87** |  |  | 142 | 136 | 125 | 123 |
| **88** |  |  | 144 | 137 | 127 | 125 |
| **89** |  |  | 145 | 138 | 128 | 126 |
| **90** |  |  | 146 | 140 | 129 | 127 |
| **91** |  |  | 147 | 141 | 130 | 129 |
| **92** |  |  | 149 | 142 | 131 | 130 |
| **93** |  |  | 150 | 144 | 132 | 131 |
| **94** |  |  |  | 145 | 133 | 132 |
| **95** |  |  |  | 146 | 134 | 134 |
| **96** |  |  |  | 147 | 136 | 135 |
| **97** |  |  |  | 149 | 137 | 136 |
| **98** |  |  |  | 150 | 138 | 138 |
| **99** |  |  |  |  | 139 | 139 |
| **100** |  |  |  |  | 140 | 140 |
| **101** |  |  |  |  | 141 | 142 |
| **102** |  |  |  |  | 142 | 143 |
| **103** |  |  |  |  | 143 | 144 |
| **104** |  |  |  |  | 144 | 146 |
| **105** |  |  |  |  | 146 | 147 |
| **106** |  |  |  |  | 147 | 148 |
| **107** |  |  |  |  | 148 | 149 |
| **108** |  |  |  |  | 149 | 151 |
| **109** |  |  |  |  | 150 |  |

| **Table B2.** Raw scores elementary school 'Moving Sideways' (MS) (Girls) | | | | | | |
| --- | --- | --- | --- | --- | --- | --- |
| **Raw Score / Age (years)** | **6-6.99** | **7-7.99** | **8-8.99** | **9-9.99** | **10-10.99** | **11-11.99** |
| **1** | 22 | 55 | 31 | 41 | 21 | 0 |
| **2** | 24 | 56 | 33 | 42 | 23 | 0 |
| **3** | 26 | 57 | 35 | 44 | 24 | 0 |
| **4** | 29 | 59 | 37 | 45 | 26 | 2 |
| **5** | 31 | 60 | 38 | 47 | 28 | 4 |
| **6** | 34 | 62 | 40 | 48 | 29 | 6 |
| **7** | 36 | 63 | 42 | 49 | 31 | 8 |
| **8** | 38 | 65 | 43 | 51 | 32 | 11 |
| **9** | 41 | 66 | 45 | 52 | 34 | 13 |
| **10** | 43 | 67 | 47 | 54 | 36 | 15 |
| **11** | 46 | 69 | 48 | 55 | 37 | 17 |
| **12** | 48 | 70 | 50 | 57 | 39 | 19 |
| **13** | 51 | 72 | 52 | 58 | 41 | 21 |
| **14** | 53 | 73 | 53 | 60 | 42 | 23 |
| **15** | 55 | 75 | 55 | 61 | 44 | 25 |
| **16** | 58 | 76 | 57 | 63 | 46 | 27 |
| **17** | 60 | 77 | 58 | 64 | 47 | 29 |
| **18** | 63 | 79 | 60 | 65 | 49 | 32 |
| **19** | 65 | 80 | 62 | 67 | 51 | 34 |
| **20** | 67 | 82 | 63 | 68 | 52 | 36 |
| **21** | 70 | 83 | 65 | 70 | 54 | 38 |
| **22** | 72 | 85 | 67 | 71 | 56 | 40 |
| **23** | 75 | 86 | 68 | 73 | 57 | 42 |
| **24** | 77 | 87 | 70 | 74 | 59 | 44 |
| **25** | 80 | 89 | 72 | 76 | 60 | 46 |
| **26** | 82 | 90 | 73 | 77 | 62 | 48 |
| **27** | 84 | 92 | 75 | 79 | 64 | 50 |
| **28** | 87 | 93 | 77 | 80 | 65 | 53 |
| **29** | 89 | 95 | 78 | 81 | 67 | 55 |
| **30** | 92 | 96 | 80 | 83 | 69 | 57 |
| **31** | 94 | 98 | 82 | 84 | 70 | 59 |
| **32** | 96 | 99 | 83 | 86 | 72 | 61 |
| **33** | 99 | 100 | 85 | 87 | 74 | 63 |
| **34** | 101 | 102 | 87 | 89 | 75 | 65 |
| **35** | 104 | 103 | 88 | 90 | 77 | 67 |
| **36** | 106 | 105 | 90 | 92 | 79 | 69 |
| **37** | 109 | 106 | 92 | 93 | 80 | 72 |
| **38** | 111 | 108 | 93 | 95 | 82 | 74 |
| **39** | 113 | 109 | 95 | 96 | 84 | 76 |
| **40** | 116 | 110 | 97 | 97 | 85 | 78 |
| **41** | 118 | 112 | 98 | 99 | 87 | 80 |
| **42** | 121 | 113 | 100 | 100 | 89 | 82 |
| **43** | 123 | 115 | 102 | 102 | 90 | 84 |
| **44** | 125 | 116 | 103 | 103 | 92 | 86 |
| **45** | 128 | 118 | 105 | 105 | 93 | 88 |
| **46** | 130 | 119 | 107 | 106 | 95 | 90 |
| **47** | 133 | 120 | 108 | 108 | 97 | 93 |
| **48** | 135 | 122 | 110 | 109 | 98 | 95 |
| **49** | 138 | 123 | 112 | 110 | 100 | 97 |
| **50** | 140 | 125 | 113 | 112 | 102 | 99 |
| **51** | 142 | 126 | 115 | 113 | 103 | 101 |
| **52** | 145 | 128 | 117 | 115 | 105 | 103 |
| **53** | 147 | 129 | 119 | 116 | 107 | 105 |
| **54** | 150 | 130 | 120 | 118 | 108 | 107 |
| **55** |  | 132 | 122 | 119 | 110 | 109 |
| **56** |  | 133 | 124 | 121 | 112 | 111 |
| **57** |  | 135 | 125 | 122 | 113 | 114 |
| **58** |  | 136 | 127 | 124 | 115 | 116 |
| **59** |  | 138 | 129 | 125 | 117 | 118 |
| **60** |  | 139 | 130 | 126 | 118 | 120 |
| **61** |  | 141 | 132 | 128 | 120 | 122 |
| **62** |  | 142 | 134 | 129 | 121 | 124 |
| **63** |  | 143 | 135 | 131 | 123 | 126 |
| **64** |  | 145 | 137 | 132 | 125 | 128 |
| **65** |  | 146 | 139 | 134 | 126 | 130 |
| **66** |  | 148 | 140 | 135 | 128 | 133 |
| **67** |  | 149 | 142 | 137 | 130 | 135 |
| **68** |  | 151 | 144 | 138 | 131 | 137 |
| **69** |  |  | 145 | 140 | 133 | 139 |
| **70** |  |  | 147 | 141 | 135 | 141 |
| **71** |  |  | 149 | 142 | 136 | 143 |
| **72** |  |  | 150 | 144 | 138 | 145 |
| **73** |  |  |  | 145 | 140 | 147 |
| **74** |  |  |  | 147 | 141 | 149 |
| **75** |  |  |  | 148 | 143 | 151 |
| **76** |  |  |  | 150 | 145 |  |
| **77** |  |  |  |  | 146 |  |
| **78** |  |  |  |  | 148 |  |
| **79** |  |  |  |  | 149 |  |
| **80** |  |  |  |  | 151 |  |

| **Table B3.** Raw scores elementary school 'Balancing Backwards' (BB) (Girls) | | | | | | |
| --- | --- | --- | --- | --- | --- | --- |
| **Raw Score / Age (years)** | **6-6.99** | **7-7.99** | **8-8.99** | **9-9.99** | **10-10.99** | **11-11.99** |
| **1** | 65 | 55 | 49 | 48 | 45 | 33 |
| **2** | 66 | 56 | 51 | 49 | 46 | 35 |
| **3** | 67 | 57 | 52 | 51 | 48 | 36 |
| **4** | 68 | 58 | 53 | 52 | 49 | 37 |
| **5** | 69 | 60 | 54 | 53 | 50 | 38 |
| **6** | 70 | 61 | 55 | 54 | 51 | 40 |
| **7** | 71 | 62 | 56 | 55 | 52 | 41 |
| **8** | 72 | 63 | 57 | 56 | 53 | 42 |
| **9** | 73 | 64 | 58 | 57 | 54 | 43 |
| **10** | 74 | 66 | 59 | 58 | 55 | 45 |
| **11** | 76 | 67 | 61 | 59 | 57 | 46 |
| **12** | 77 | 68 | 62 | 60 | 58 | 47 |
| **13** | 78 | 69 | 63 | 61 | 59 | 48 |
| **14** | 79 | 71 | 64 | 62 | 60 | 50 |
| **15** | 80 | 72 | 65 | 63 | 61 | 51 |
| **16** | 81 | 73 | 66 | 65 | 62 | 52 |
| **17** | 82 | 74 | 67 | 66 | 63 | 53 |
| **18** | 83 | 75 | 68 | 67 | 64 | 55 |
| **19** | 84 | 77 | 69 | 68 | 66 | 56 |
| **20** | 85 | 78 | 70 | 69 | 67 | 57 |
| **21** | 86 | 79 | 72 | 70 | 68 | 58 |
| **22** | 88 | 80 | 73 | 71 | 69 | 60 |
| **23** | 89 | 81 | 74 | 72 | 70 | 61 |
| **24** | 90 | 83 | 75 | 73 | 71 | 62 |
| **25** | 91 | 84 | 76 | 74 | 72 | 63 |
| **26** | 92 | 85 | 77 | 75 | 73 | 65 |
| **27** | 93 | 86 | 78 | 76 | 75 | 66 |
| **28** | 94 | 87 | 79 | 77 | 76 | 67 |
| **29** | 95 | 89 | 80 | 79 | 77 | 68 |
| **30** | 96 | 90 | 82 | 80 | 78 | 70 |
| **31** | 97 | 91 | 83 | 81 | 79 | 71 |
| **32** | 99 | 92 | 84 | 82 | 80 | 72 |
| **33** | 100 | 93 | 85 | 83 | 81 | 73 |
| **34** | 101 | 95 | 86 | 84 | 82 | 75 |
| **35** | 102 | 96 | 87 | 85 | 84 | 76 |
| **36** | 103 | 97 | 88 | 86 | 85 | 77 |
| **37** | 104 | 98 | 89 | 87 | 86 | 78 |
| **38** | 105 | 99 | 90 | 88 | 87 | 80 |
| **39** | 106 | 101 | 91 | 89 | 88 | 81 |
| **40** | 107 | 102 | 93 | 90 | 89 | 82 |
| **41** | 108 | 103 | 94 | 91 | 90 | 83 |
| **42** | 110 | 104 | 95 | 93 | 91 | 85 |
| **43** | 111 | 105 | 96 | 94 | 92 | 86 |
| **44** | 112 | 107 | 97 | 95 | 94 | 87 |
| **45** | 113 | 108 | 98 | 96 | 95 | 88 |
| **46** | 114 | 109 | 99 | 97 | 96 | 90 |
| **47** | 115 | 110 | 100 | 98 | 97 | 91 |
| **48** | 116 | 111 | 101 | 99 | 98 | 92 |
| **49** | 117 | 113 | 103 | 100 | 99 | 93 |
| **50** | 118 | 114 | 104 | 101 | 100 | 95 |
| **51** | 119 | 115 | 105 | 102 | 101 | 96 |
| **52** | 120 | 116 | 106 | 103 | 103 | 97 |
| **53** | 122 | 117 | 107 | 104 | 104 | 98 |
| **54** | 123 | 119 | 108 | 105 | 105 | 100 |
| **55** | 124 | 120 | 109 | 107 | 106 | 101 |
| **56** | 125 | 121 | 110 | 108 | 107 | 102 |
| **57** | 126 | 122 | 111 | 109 | 108 | 103 |
| **58** | 127 | 123 | 112 | 110 | 109 | 105 |
| **59** | 128 | 125 | 114 | 111 | 110 | 106 |
| **60** | 129 | 126 | 115 | 112 | 112 | 107 |
| **61** | 130 | 127 | 116 | 113 | 113 | 108 |
| **62** | 131 | 128 | 117 | 114 | 114 | 110 |
| **63** | 133 | 129 | 118 | 115 | 115 | 111 |
| **64** | 134 | 131 | 119 | 116 | 116 | 112 |
| **65** | 135 | 132 | 120 | 117 | 117 | 113 |
| **66** | 136 | 133 | 121 | 118 | 118 | 115 |
| **67** | 137 | 134 | 122 | 119 | 119 | 116 |
| **68** | 138 | 135 | 124 | 121 | 121 | 117 |
| **69** | 139 | 137 | 125 | 122 | 122 | 118 |
| **70** | 140 | 138 | 126 | 123 | 123 | 120 |
| **71** | 141 | 139 | 127 | 124 | 124 | 121 |
| **72** | 142 | 140 | 128 | 125 | 125 | 122 |
| **Table B4.** Raw scores elementary school 'Eye-Hand Coordination' (EHC) (Girls) | | | | | | |
| **Raw Score / Age (years)** | **6-6.99** | **7-7.99** | **8-8.99** | **9-9.99** | **10-10.99** | **11-11.99** |
| **0** | 94 | 91 | 89 | 81 | 81 | 78 |
| **1** | 100 | 94 | 91 | 83 | 83 | 79 |
| **2** | 105 | 97 | 93 | 84 | 84 | 80 |
| **3** | 110 | 100 | 94 | 85 | 85 | 81 |
| **4** | 115 | 103 | 96 | 87 | 86 | 82 |
| **5** | 121 | 105 | 98 | 88 | 87 | 83 |
| **6** | 126 | 108 | 99 | 90 | 88 | 83 |
| **7** | 131 | 111 | 101 | 91 | 89 | 84 |
| **8** | 136 | 114 | 103 | 93 | 90 | 85 |
| **9** | 142 | 117 | 105 | 94 | 91 | 86 |
| **10** | 147 | 119 | 106 | 95 | 92 | 87 |
| **11** | 152 | 122 | 108 | 97 | 93 | 88 |
| **12** |  | 125 | 110 | 98 | 94 | 89 |
| **13** |  | 128 | 111 | 100 | 95 | 90 |
| **14** |  | 131 | 113 | 101 | 97 | 91 |
| **15** |  | 133 | 115 | 102 | 98 | 92 |
| **16** |  | 136 | 117 | 104 | 99 | 93 |
| **17** |  | 139 | 118 | 105 | 100 | 94 |
| **18** |  | 142 | 120 | 107 | 101 | 95 |
| **19** |  | 145 | 122 | 108 | 102 | 96 |
| **20** |  | 148 | 123 | 110 | 103 | 96 |
| **21** |  | 150 | 125 | 111 | 104 | 97 |
| **22** |  |  | 127 | 112 | 105 | 98 |
| **23** |  |  | 128 | 114 | 106 | 99 |
| **24** |  |  | 130 | 115 | 107 | 100 |
| **25** |  |  | 132 | 117 | 108 | 101 |
| **26** |  |  | 134 | 118 | 110 | 102 |
| **27** |  |  | 135 | 120 | 111 | 103 |
| **28** |  |  | 137 | 121 | 112 | 104 |
| **29** |  |  | 139 | 122 | 113 | 105 |
| **30** |  |  | 140 | 124 | 114 | 106 |
| **31** |  |  | 142 | 125 | 115 | 107 |
| **32** |  |  | 144 | 127 | 116 | 108 |
| **33** |  |  | 146 | 128 | 117 | 109 |
| **34** |  |  | 147 | 130 | 118 | 109 |
| **35** |  |  | 149 | 131 | 119 | 110 |
| **36** |  |  | 151 | 132 | 120 | 111 |
| **37** |  |  |  | 134 | 121 | 112 |
| **38** |  |  |  | 135 | 122 | 113 |
| **39** |  |  |  | 137 | 124 | 114 |
| **40** |  |  |  | 138 | 125 | 115 |
| **41** |  |  |  | 139 | 126 | 116 |
| **42** |  |  |  | 141 | 127 | 117 |
| **43** |  |  |  | 142 | 128 | 118 |
| **44** |  |  |  | 144 | 129 | 119 |
| **45** |  |  |  | 145 | 130 | 120 |
| **46** |  |  |  | 147 | 131 | 121 |
| **47** |  |  |  | 148 | 132 | 122 |
| **48** |  |  |  | 149 | 133 | 122 |
| **49** |  |  |  | 151 | 134 | 123 |
| **50** |  |  |  |  | 135 | 124 |
| **51** |  |  |  |  | 137 | 125 |
| **52** |  |  |  |  | 138 | 126 |
| **53** |  |  |  |  | 139 | 127 |
| **54** |  |  |  |  | 140 | 128 |
| **55** |  |  |  |  | 141 | 129 |
| **56** |  |  |  |  | 142 | 130 |
| **57** |  |  |  |  | 143 | 131 |
| **58** |  |  |  |  | 144 | 132 |
| **59** |  |  |  |  | 145 | 133 |
| **60** |  |  |  |  | 146 | 134 |
| **61** |  |  |  |  | 147 | 134 |
| **62** |  |  |  |  | 148 | 135 |
| **63** |  |  |  |  | 150 | 136 |
| **64** |  |  |  |  |  | 137 |
| **65** |  |  |  |  |  | 138 |
| **66** |  |  |  |  |  | 139 |
| **67** |  |  |  |  |  | 140 |
| **68** |  |  |  |  |  | 141 |
| **69** |  |  |  |  |  | 142 |
| **70** |  |  |  |  |  | 143 |
| **71** |  |  |  |  |  | 144 |
| **72** |  |  |  |  |  | 145 |
| **73** |  |  |  |  |  | 146 |
| **74** |  |  |  |  |  | 147 |
| **75** |  |  |  |  |  | 147 |
| **76** |  |  |  |  |  | 148 |
| **77** |  |  |  |  |  | 149 |
| **78** |  |  |  |  |  | 150 |

| **Table B5.** Raw scores elementary school 'Jumping Sideways' (JS) (Boys) | | | | | | |
| --- | --- | --- | --- | --- | --- | --- |
| **Raw Score / Age (years)** | **6-6.99** | **7-7.99** | **8-8.99** | **9-9.99** | **10-10.99** | **11-11.99** |
| **1** | 43 | 28 | 15 | 22 | 7 | 11 |
| **2** | 44 | 29 | 17 | 23 | 8 | 12 |
| **3** | 46 | 31 | 19 | 24 | 10 | 13 |
| **4** | 47 | 32 | 20 | 26 | 11 | 15 |
| **5** | 48 | 34 | 22 | 27 | 12 | 16 |
| **6** | 50 | 35 | 23 | 28 | 14 | 17 |
| **7** | 51 | 37 | 25 | 29 | 15 | 18 |
| **8** | 53 | 38 | 26 | 31 | 17 | 20 |
| **9** | 54 | 40 | 28 | 32 | 18 | 21 |
| **10** | 55 | 41 | 29 | 33 | 19 | 22 |
| **11** | 57 | 43 | 31 | 34 | 21 | 23 |
| **12** | 58 | 44 | 32 | 35 | 22 | 25 |
| **13** | 60 | 46 | 34 | 37 | 23 | 26 |
| **14** | 61 | 47 | 36 | 38 | 25 | 27 |
| **15** | 62 | 48 | 37 | 39 | 26 | 28 |
| **16** | 64 | 50 | 39 | 40 | 28 | 29 |
| **17** | 65 | 51 | 40 | 42 | 29 | 31 |
| **18** | 67 | 53 | 42 | 43 | 30 | 32 |
| **19** | 68 | 54 | 43 | 44 | 32 | 33 |
| **20** | 69 | 56 | 45 | 45 | 33 | 34 |
| **21** | 71 | 57 | 46 | 47 | 35 | 36 |
| **22** | 72 | 59 | 48 | 48 | 36 | 37 |
| **23** | 74 | 60 | 49 | 49 | 37 | 38 |
| **24** | 75 | 62 | 51 | 50 | 39 | 39 |
| **25** | 76 | 63 | 53 | 51 | 40 | 41 |
| **26** | 78 | 65 | 54 | 53 | 42 | 42 |
| **27** | 79 | 66 | 56 | 54 | 43 | 43 |
| **28** | 81 | 68 | 57 | 55 | 44 | 44 |
| **29** | 82 | 69 | 59 | 56 | 46 | 45 |
| **30** | 83 | 71 | 60 | 58 | 47 | 47 |
| **31** | 85 | 72 | 62 | 59 | 49 | 48 |
| **32** | 86 | 74 | 63 | 60 | 50 | 49 |
| **33** | 88 | 75 | 65 | 61 | 51 | 50 |
| **34** | 89 | 77 | 66 | 62 | 53 | 52 |
| **35** | 90 | 78 | 68 | 64 | 54 | 53 |
| **36** | 92 | 80 | 70 | 65 | 56 | 54 |
| **37** | 93 | 81 | 71 | 66 | 57 | 55 |
| **38** | 95 | 82 | 73 | 67 | 58 | 57 |
| **39** | 96 | 84 | 74 | 69 | 60 | 58 |
| **40** | 97 | 85 | 76 | 70 | 61 | 59 |
| **41** | 99 | 87 | 77 | 71 | 63 | 60 |
| **42** | 100 | 88 | 79 | 72 | 64 | 61 |
| **43** | 102 | 90 | 80 | 74 | 65 | 63 |
| **44** | 103 | 91 | 82 | 75 | 67 | 64 |
| **45** | 104 | 93 | 83 | 76 | 68 | 65 |
| **46** | 106 | 94 | 85 | 77 | 70 | 66 |
| **47** | 107 | 96 | 87 | 78 | 71 | 68 |
| **48** | 109 | 97 | 88 | 80 | 72 | 69 |
| **49** | 110 | 99 | 90 | 81 | 74 | 70 |
| **50** | 111 | 100 | 91 | 82 | 75 | 71 |
| **51** | 113 | 102 | 93 | 83 | 77 | 73 |
| **52** | 114 | 103 | 94 | 85 | 78 | 74 |
| **53** | 116 | 105 | 96 | 86 | 79 | 75 |
| **54** | 117 | 106 | 97 | 87 | 81 | 76 |
| **55** | 118 | 108 | 99 | 88 | 82 | 78 |
| **56** | 120 | 109 | 100 | 90 | 83 | 79 |
| **57** | 121 | 111 | 102 | 91 | 85 | 80 |
| **58** | 123 | 112 | 104 | 92 | 86 | 81 |
| **59** | 124 | 113 | 105 | 93 | 88 | 82 |
| **60** | 125 | 115 | 107 | 94 | 89 | 84 |
| **61** | 127 | 116 | 108 | 96 | 90 | 85 |
| **62** | 128 | 118 | 110 | 97 | 92 | 86 |
| **63** | 130 | 119 | 111 | 98 | 93 | 87 |
| **64** | 131 | 121 | 113 | 99 | 95 | 89 |
| **65** | 132 | 122 | 114 | 101 | 96 | 90 |
| **66** | 134 | 124 | 116 | 102 | 97 | 91 |
| **67** | 135 | 125 | 117 | 103 | 99 | 92 |
| **68** | 137 | 127 | 119 | 104 | 100 | 94 |
| **69** | 138 | 128 | 121 | 105 | 102 | 95 |
| **70** | 139 | 130 | 122 | 107 | 103 | 96 |
| **71** | 141 | 131 | 124 | 108 | 104 | 97 |
| **72** | 142 | 133 | 125 | 109 | 106 | 98 |
| **73** | 144 | 134 | 127 | 110 | 107 | 100 |
| **74** | 145 | 136 | 128 | 112 | 109 | 101 |
| **75** | 146 | 137 | 130 | 113 | 110 | 102 |
| **76** | 148 | 139 | 131 | 114 | 111 | 103 |
| **77** | 149 | 140 | 133 | 115 | 113 | 105 |
| **78** | 151 | 142 | 134 | 117 | 114 | 106 |
| **79** |  | 143 | 136 | 118 | 116 | 107 |
| **80** |  | 145 | 138 | 119 | 117 | 108 |
| **81** |  | 146 | 139 | 120 | 118 | 110 |
| **82** |  | 147 | 141 | 121 | 120 | 111 |
| **83** |  | 149 | 142 | 123 | 121 | 112 |
| **84** |  | 150 | 144 | 124 | 123 | 113 |
| **85** |  |  | 145 | 125 | 124 | 114 |
| **86** |  |  | 147 | 126 | 125 | 116 |
| **87** |  |  | 148 | 128 | 127 | 117 |
| **88** |  |  | 150 | 129 | 128 | 118 |
| **89** |  |  |  | 130 | 130 | 119 |
| **90** |  |  |  | 131 | 131 | 121 |
| **91** |  |  |  | 132 | 132 | 122 |
| **92** |  |  |  | 134 | 134 | 123 |
| **93** |  |  |  | 135 | 135 | 124 |
| **94** |  |  |  | 136 | 137 | 126 |
| **95** |  |  |  | 137 | 138 | 127 |
| **96** |  |  |  | 139 | 139 | 128 |
| **97** |  |  |  | 140 | 141 | 129 |
| **98** |  |  |  | 141 | 142 | 130 |
| **99** |  |  |  | 142 | 143 | 132 |
| **100** |  |  |  | 144 | 145 | 133 |
| **101** |  |  |  | 145 | 146 | 134 |
| **102** |  |  |  | 146 | 148 | 135 |
| **103** |  |  |  | 147 | 149 | 137 |
| **104** |  |  |  | 148 | 150 | 138 |
| **105** |  |  |  | 150 |  | 139 |
| **106** |  |  |  |  |  | 140 |
| **107** |  |  |  |  |  | 142 |
| **108** |  |  |  |  |  | 143 |
| **109** |  |  |  |  |  | 144 |
| **110** |  |  |  |  |  | 145 |
| **111** |  |  |  |  |  | 146 |
| **112** |  |  |  |  |  | 148 |
| **113** |  |  |  |  |  | 149 |
| **114** |  |  |  |  |  | 150 |

| **Table B6.** Raw scores elementary school 'Moving Sideways' (MS) (Boys) | | | | | | |
| --- | --- | --- | --- | --- | --- | --- |
| **Raw Score / Age (years)** | **6-6.99** | **7-7.99** | **8-8.99** | **9-9.99** | **10-10.99** | **11-11.99** |
| **1** | 27 | 56 | 39 | 29 | 1 | 25 |
| **2** | 29 | 58 | 40 | 30 | 3 | 27 |
| **3** | 31 | 59 | 42 | 32 | 5 | 28 |
| **4** | 34 | 60 | 43 | 34 | 7 | 30 |
| **5** | 36 | 61 | 45 | 35 | 9 | 31 |
| **6** | 38 | 63 | 46 | 37 | 12 | 33 |
| **7** | 40 | 64 | 48 | 38 | 14 | 34 |
| **8** | 42 | 65 | 49 | 40 | 16 | 36 |
| **9** | 44 | 67 | 51 | 42 | 18 | 37 |
| **10** | 46 | 68 | 52 | 43 | 20 | 39 |
| **11** | 48 | 69 | 54 | 45 | 22 | 40 |
| **12** | 50 | 70 | 55 | 46 | 24 | 42 |
| **13** | 53 | 72 | 57 | 48 | 26 | 44 |
| **14** | 55 | 73 | 58 | 50 | 28 | 45 |
| **15** | 57 | 74 | 60 | 51 | 30 | 47 |
| **16** | 59 | 75 | 61 | 53 | 32 | 48 |
| **17** | 61 | 77 | 63 | 54 | 34 | 50 |
| **18** | 63 | 78 | 64 | 56 | 36 | 51 |
| **19** | 65 | 79 | 66 | 58 | 38 | 53 |
| **20** | 67 | 80 | 67 | 59 | 41 | 54 |
| **21** | 69 | 82 | 69 | 61 | 43 | 56 |
| **22** | 72 | 83 | 70 | 63 | 45 | 57 |
| **23** | 74 | 84 | 72 | 64 | 47 | 59 |
| **24** | 76 | 86 | 73 | 66 | 49 | 60 |
| **25** | 78 | 87 | 75 | 67 | 51 | 62 |
| **26** | 80 | 88 | 76 | 69 | 53 | 63 |
| **27** | 82 | 89 | 78 | 71 | 55 | 65 |
| **28** | 84 | 91 | 79 | 72 | 57 | 67 |
| **29** | 86 | 92 | 81 | 74 | 59 | 68 |
| **30** | 88 | 93 | 82 | 75 | 61 | 70 |
| **31** | 91 | 94 | 84 | 77 | 63 | 71 |
| **32** | 93 | 96 | 85 | 79 | 65 | 73 |
| **33** | 95 | 97 | 87 | 80 | 67 | 74 |
| **34** | 97 | 98 | 88 | 82 | 69 | 76 |
| **35** | 99 | 99 | 90 | 83 | 72 | 77 |
| **36** | 101 | 101 | 92 | 85 | 74 | 79 |
| **37** | 103 | 102 | 93 | 87 | 76 | 80 |
| **38** | 105 | 103 | 95 | 88 | 78 | 82 |
| **39** | 107 | 105 | 96 | 90 | 80 | 83 |
| **40** | 110 | 106 | 98 | 91 | 82 | 85 |
| **41** | 112 | 107 | 99 | 93 | 84 | 86 |
| **42** | 114 | 108 | 101 | 95 | 86 | 88 |
| **43** | 116 | 110 | 102 | 96 | 88 | 90 |
| **44** | 118 | 111 | 104 | 98 | 90 | 91 |
| **45** | 120 | 112 | 105 | 99 | 92 | 93 |
| **46** | 122 | 113 | 107 | 101 | 94 | 94 |
| **47** | 124 | 115 | 108 | 103 | 96 | 96 |
| **48** | 126 | 116 | 110 | 104 | 98 | 97 |
| **49** | 129 | 117 | 111 | 106 | 101 | 99 |
| **50** | 131 | 118 | 113 | 107 | 103 | 100 |
| **51** | 133 | 120 | 114 | 109 | 105 | 102 |
| **52** | 135 | 121 | 116 | 111 | 107 | 103 |
| **53** | 137 | 122 | 117 | 112 | 109 | 105 |
| **54** | 139 | 124 | 119 | 114 | 111 | 106 |
| **55** | 141 | 125 | 120 | 115 | 113 | 108 |
| **56** | 143 | 126 | 122 | 117 | 115 | 109 |
| **57** | 146 | 127 | 123 | 119 | 117 | 111 |
| **58** | 148 | 129 | 125 | 120 | 119 | 113 |
| **59** | 150 | 130 | 126 | 122 | 121 | 114 |
| **60** |  | 131 | 128 | 123 | 123 | 116 |
| **61** |  | 132 | 129 | 125 | 125 | 117 |
| **62** |  | 134 | 131 | 127 | 127 | 119 |
| **63** |  | 135 | 132 | 128 | 129 | 120 |
| **64** |  | 136 | 134 | 130 | 132 | 122 |
| **65** |  | 137 | 135 | 131 | 134 | 123 |
| **66** |  | 139 | 137 | 133 | 136 | 125 |
| **67** |  | 140 | 138 | 135 | 138 | 126 |
| **68** |  | 141 | 140 | 136 | 140 | 128 |
| **69** |  | 143 | 141 | 138 | 142 | 129 |
| **70** |  | 144 | 143 | 139 | 144 | 131 |
| **71** |  | 145 | 144 | 141 | 146 | 132 |
| **72** |  | 146 | 146 | 143 | 148 | 134 |
| **73** |  | 148 | 147 | 144 | 150 | 136 |
| **74** |  | 149 | 149 | 146 |  | 137 |
| **75** |  | 150 | 150 | 147 |  | 139 |
| **76** |  |  |  | 149 |  | 140 |
| **77** |  |  |  | 151 |  | 142 |
| **78** |  |  |  |  |  | 143 |
| **79** |  |  |  |  |  | 145 |
| **80** |  |  |  |  |  | 146 |
| **81** |  |  |  |  |  | 148 |
| **82** |  |  |  |  |  | 149 |
| **83** |  |  |  |  |  | 151 |

| **Table B7.** Raw scores elementary school 'Balancing Backwards' (BB) (Boys) | | | | | | |
| --- | --- | --- | --- | --- | --- | --- |
| **Raw Score / Age (years)** | **6-6.99** | **7-7.99** | **8-8.99** | **9-9.99** | **10-10.99** | **11-11.99** |
| **1** | 61 | 52 | 50 | 50 | 42 | 42 |
| **2** | 63 | 54 | 51 | 51 | 43 | 44 |
| **3** | 64 | 55 | 52 | 52 | 44 | 45 |
| **4** | 65 | 56 | 53 | 53 | 45 | 46 |
| **5** | 67 | 58 | 54 | 55 | 47 | 47 |
| **6** | 68 | 59 | 56 | 56 | 48 | 48 |
| **7** | 70 | 60 | 57 | 57 | 49 | 49 |
| **8** | 71 | 62 | 58 | 58 | 50 | 50 |
| **9** | 73 | 63 | 59 | 59 | 52 | 52 |
| **10** | 74 | 64 | 60 | 60 | 53 | 53 |
| **11** | 76 | 66 | 62 | 61 | 54 | 54 |
| **12** | 77 | 67 | 63 | 62 | 56 | 55 |
| **13** | 79 | 69 | 64 | 64 | 57 | 56 |
| **14** | 80 | 70 | 65 | 65 | 58 | 57 |
| **15** | 82 | 71 | 66 | 66 | 59 | 58 |
| **16** | 83 | 73 | 68 | 67 | 61 | 60 |
| **17** | 84 | 74 | 69 | 68 | 62 | 61 |
| **18** | 86 | 75 | 70 | 69 | 63 | 62 |
| **19** | 87 | 77 | 71 | 70 | 64 | 63 |
| **20** | 89 | 78 | 73 | 71 | 66 | 64 |
| **21** | 90 | 79 | 74 | 72 | 67 | 65 |
| **22** | 92 | 81 | 75 | 74 | 68 | 66 |
| **23** | 93 | 82 | 76 | 75 | 70 | 67 |
| **24** | 95 | 84 | 77 | 76 | 71 | 69 |
| **25** | 96 | 85 | 79 | 77 | 72 | 70 |
| **26** | 98 | 86 | 80 | 78 | 73 | 71 |
| **27** | 99 | 88 | 81 | 79 | 75 | 72 |
| **28** | 101 | 89 | 82 | 80 | 76 | 73 |
| **29** | 102 | 90 | 83 | 81 | 77 | 74 |
| **30** | 104 | 92 | 85 | 82 | 78 | 75 |
| **31** | 105 | 93 | 86 | 84 | 80 | 77 |
| **32** | 106 | 94 | 87 | 85 | 81 | 78 |
| **33** | 108 | 96 | 88 | 86 | 82 | 79 |
| **34** | 109 | 97 | 89 | 87 | 84 | 80 |
| **35** | 111 | 98 | 91 | 88 | 85 | 81 |
| **36** | 112 | 100 | 92 | 89 | 86 | 82 |
| **37** | 114 | 101 | 93 | 90 | 87 | 83 |
| **38** | 115 | 103 | 94 | 91 | 89 | 85 |
| **39** | 117 | 104 | 96 | 93 | 90 | 86 |
| **40** | 118 | 105 | 97 | 94 | 91 | 87 |
| **41** | 120 | 107 | 98 | 95 | 92 | 88 |
| **42** | 121 | 108 | 99 | 96 | 94 | 89 |
| **43** | 123 | 109 | 100 | 97 | 95 | 90 |
| **44** | 124 | 111 | 102 | 98 | 96 | 91 |
| **45** | 125 | 112 | 103 | 99 | 97 | 92 |
| **46** | 127 | 113 | 104 | 100 | 99 | 94 |
| **47** | 128 | 115 | 105 | 101 | 100 | 95 |
| **48** | 130 | 116 | 106 | 103 | 101 | 96 |
| **49** | 131 | 118 | 108 | 104 | 103 | 97 |
| **50** | 133 | 119 | 109 | 105 | 104 | 98 |
| **51** | 134 | 120 | 110 | 106 | 105 | 99 |
| **52** | 136 | 122 | 111 | 107 | 106 | 100 |
| **53** | 137 | 123 | 112 | 108 | 108 | 102 |
| **54** | 139 | 124 | 114 | 109 | 109 | 103 |
| **55** | 140 | 126 | 115 | 110 | 110 | 104 |
| **56** | 142 | 127 | 116 | 112 | 111 | 105 |
| **57** | 143 | 128 | 117 | 113 | 113 | 106 |
| **58** | 144 | 130 | 119 | 114 | 114 | 107 |
| **59** | 146 | 131 | 120 | 115 | 115 | 108 |
| **60** | 147 | 132 | 121 | 116 | 117 | 110 |
| **61** | 149 | 134 | 122 | 117 | 118 | 111 |
| **62** | 150 | 135 | 123 | 118 | 119 | 112 |
| **63** | 152 | 137 | 125 | 119 | 120 | 113 |
| **64** | 153 | 138 | 126 | 120 | 122 | 114 |
| **65** | 155 | 139 | 127 | 122 | 123 | 115 |
| **66** | 156 | 141 | 128 | 123 | 124 | 116 |
| **67** | 158 | 142 | 129 | 124 | 125 | 117 |
| **68** | 159 | 143 | 131 | 125 | 127 | 119 |
| **69** | 161 | 145 | 132 | 126 | 128 | 120 |
| **70** | 162 | 146 | 133 | 127 | 129 | 121 |
| **71** | 164 | 147 | 134 | 128 | 130 | 122 |
| **72** | 165 | 149 | 135 | 129 | 132 | 123 |

| **Table B8.** Raw scores elementary school 'Eye-Hand Coordination' (EHC) (Boys) | | | | | | |
| --- | --- | --- | --- | --- | --- | --- |
| **Raw Score / Age (years)** | **6-6.99** | **7-7.99** | **8-8.99** | **9-9.99** | **10-10.99** | **11-11.99** |
| **0** | 92 | 87 | 81 | 75 | 63 | 50 |
| **1** | 94 | 88 | 82 | 76 | 64 | 51 |
| **2** | 96 | 90 | 83 | 77 | 65 | 52 |
| **3** | 98 | 91 | 84 | 78 | 66 | 54 |
| **4** | 100 | 93 | 85 | 79 | 67 | 55 |
| **5** | 102 | 94 | 86 | 80 | 68 | 56 |
| **6** | 104 | 96 | 87 | 81 | 69 | 58 |
| **7** | 106 | 97 | 88 | 82 | 70 | 59 |
| **8** | 109 | 99 | 89 | 83 | 71 | 60 |
| **9** | 111 | 100 | 90 | 84 | 73 | 62 |
| **10** | 113 | 102 | 91 | 85 | 74 | 63 |
| **11** | 115 | 103 | 92 | 86 | 75 | 65 |
| **12** | 117 | 104 | 93 | 87 | 76 | 66 |
| **13** | 119 | 106 | 94 | 88 | 77 | 67 |
| **14** | 121 | 107 | 95 | 89 | 78 | 69 |
| **15** | 124 | 109 | 96 | 90 | 79 | 70 |
| **16** | 126 | 110 | 97 | 91 | 80 | 71 |
| **17** | 128 | 112 | 98 | 92 | 81 | 73 |
| **18** | 130 | 113 | 99 | 93 | 82 | 74 |
| **19** | 132 | 115 | 100 | 94 | 83 | 76 |
| **20** | 134 | 116 | 101 | 95 | 84 | 77 |
| **21** | 136 | 118 | 102 | 96 | 85 | 78 |
| **22** | 139 | 119 | 103 | 97 | 86 | 80 |
| **23** | 141 | 121 | 104 | 98 | 87 | 81 |
| **24** | 143 | 122 | 105 | 99 | 88 | 82 |
| **25** | 145 | 124 | 106 | 100 | 89 | 84 |
| **26** | 147 | 125 | 107 | 101 | 91 | 85 |
| **27** | 149 | 127 | 108 | 102 | 92 | 86 |
| **28** | 151 | 128 | 109 | 103 | 93 | 88 |
| **29** |  | 130 | 110 | 103 | 94 | 89 |
| **30** |  | 131 | 111 | 104 | 95 | 91 |
| **31** |  | 133 | 112 | 105 | 96 | 92 |
| **32** |  | 134 | 113 | 106 | 97 | 93 |
| **33** |  | 136 | 114 | 107 | 98 | 95 |
| **34** |  | 137 | 115 | 108 | 99 | 96 |
| **35** |  | 139 | 116 | 109 | 100 | 97 |
| **36** |  | 140 | 117 | 110 | 101 | 99 |
| **37** |  | 142 | 118 | 111 | 102 | 100 |
| **38** |  | 143 | 119 | 112 | 103 | 101 |
| **39** |  | 145 | 120 | 113 | 104 | 103 |
| **40** |  | 146 | 121 | 114 | 105 | 104 |
| **41** |  | 148 | 122 | 115 | 106 | 106 |
| **42** |  | 149 | 123 | 116 | 107 | 107 |
| **43** |  | 151 | 124 | 117 | 109 | 108 |
| **44** |  |  | 125 | 118 | 110 | 110 |
| **45** |  |  | 126 | 119 | 111 | 111 |
| **46** |  |  | 127 | 120 | 112 | 112 |
| **47** |  |  | 129 | 121 | 113 | 114 |
| **48** |  |  | 130 | 122 | 114 | 115 |
| **49** |  |  | 131 | 123 | 115 | 116 |
| **50** |  |  | 132 | 124 | 116 | 118 |
| **51** |  |  | 133 | 125 | 117 | 119 |
| **52** |  |  | 134 | 126 | 118 | 121 |
| **53** |  |  | 135 | 127 | 119 | 122 |
| **54** |  |  | 136 | 128 | 120 | 123 |
| **55** |  |  | 137 | 129 | 121 | 125 |
| **56** |  |  | 138 | 130 | 122 | 126 |
| **57** |  |  | 139 | 131 | 123 | 127 |
| **58** |  |  | 140 | 132 | 124 | 129 |
| **59** |  |  | 141 | 133 | 125 | 130 |
| **60** |  |  | 142 | 134 | 127 | 132 |
| **61** |  |  | 143 | 135 | 128 | 133 |
| **62** |  |  | 144 | 136 | 129 | 134 |
| **63** |  |  | 145 | 137 | 130 | 136 |
| **64** |  |  | 146 | 138 | 131 | 137 |
| **65** |  |  | 147 | 139 | 132 | 138 |
| **66** |  |  | 148 | 140 | 133 | 140 |
| **67** |  |  | 149 | 141 | 134 | 141 |
| **68** |  |  | 150 | 142 | 135 | 142 |
| **69** |  |  |  | 143 | 136 | 144 |
| **70** |  |  |  | 144 | 137 | 145 |
| **71** |  |  |  | 145 | 138 | 147 |
| **72** |  |  |  | 146 | 139 | 148 |
| **73** |  |  |  | 147 | 140 | 149 |
| **74** |  |  |  | 148 | 141 | 151 |
| **75** |  |  |  | 149 | 142 |  |
| **76** |  |  |  | 150 | 143 |  |
| **77** |  |  |  |  | 145 |  |
| **78** |  |  |  |  | 146 |  |
| **79** |  |  |  |  | 147 |  |
| **80** |  |  |  |  | 148 |  |
| **81** |  |  |  |  | 149 |  |
| **82** |  |  |  |  | 150 |  |
