# Supplementary material for: Validation of a Motor Competence Assessment Tool for Children and Adolescents (KTK3+) With Normative Values for 6- to 19-Year-Olds
Source: Front Physiol. 2021 Jun 23;12:652952. doi: 10.3389/fphys.2021.652952 (PMC8260948; doi:10.3389/fphys.2021.652952)
Supplement: Supplementary file 1 [file Data_Sheet_1.ZIP › Supplementary_Material_Table_C.docx]

Supplementary Material

Turning tables were made for the four tests of the KTK3+, together with a table to calculate the total MQ-score. The tables are separately for children and adolescents, due to influences of maturation in the older ages.

Tables C1 to C8 display the raw performance scores for girls and boys on the JS, MS, BB and EHC task of the KTK3+ for adolescents.

| **Table C1.** Raw scores secondary school 'Jumping Sideways' (JS) (Girls) | | | | |
| --- | --- | --- | --- | --- |
| **Raw Score / Age (years)** | **12-13.99** | **14-15.99** | **16-17.99** | **18-19.99** |
| **1** | 6 | 9 | 0 | 0 |
| **2** | 7 | 10 | 0 | 0 |
| **3** | 8 | 11 | 0 | 0 |
| **4** | 10 | 12 | 0 | 0 |
| **5** | 11 | 14 | 0 | 0 |
| **6** | 12 | 15 | 0 | 0 |
| **7** | 13 | 16 | 0 | 0 |
| **8** | 15 | 17 | 0 | 0 |
| **9** | 16 | 18 | 0 | 0 |
| **10** | 17 | 19 | 0 | 0 |
| **11** | 18 | 21 | 0 | 0 |
| **12** | 20 | 22 | 0 | 0 |
| **13** | 21 | 23 | 0 | 0 |
| **14** | 22 | 24 | 0 | 0 |
| **15** | 24 | 25 | 0 | 0 |
| **16** | 25 | 26 | 0 | 0 |
| **17** | 26 | 28 | 1 | 0 |
| **18** | 27 | 29 | 2 | 0 |
| **19** | 29 | 30 | 4 | 0 |
| **20** | 30 | 31 | 5 | 0 |
| **21** | 31 | 32 | 7 | 0 |
| **22** | 32 | 34 | 8 | 1 |
| **23** | 34 | 35 | 10 | 3 |
| **24** | 35 | 36 | 11 | 4 |
| **25** | 36 | 37 | 13 | 6 |
| **26** | 38 | 38 | 14 | 7 |
| **27** | 39 | 39 | 16 | 8 |
| **28** | 40 | 41 | 17 | 10 |
| **29** | 41 | 42 | 19 | 11 |
| **30** | 43 | 43 | 20 | 13 |
| **31** | 44 | 44 | 22 | 14 |
| **32** | 45 | 45 | 23 | 16 |
| **33** | 46 | 47 | 25 | 17 |
| **34** | 48 | 48 | 26 | 19 |
| **35** | 49 | 49 | 28 | 20 |
| **36** | 50 | 50 | 29 | 22 |
| **37** | 52 | 51 | 30 | 23 |
| **38** | 53 | 52 | 32 | 25 |
| **39** | 54 | 54 | 33 | 26 |
| **40** | 55 | 55 | 35 | 28 |
| **41** | 57 | 56 | 36 | 29 |
| **42** | 58 | 57 | 38 | 31 |
| **43** | 59 | 58 | 39 | 32 |
| **44** | 60 | 60 | 41 | 34 |
| **45** | 62 | 61 | 42 | 35 |
| **46** | 63 | 62 | 44 | 37 |
| **47** | 64 | 63 | 45 | 38 |
| **48** | 66 | 64 | 47 | 39 |
| **49** | 67 | 65 | 48 | 41 |
| **50** | 68 | 67 | 50 | 42 |
| **51** | 69 | 68 | 51 | 44 |
| **52** | 71 | 69 | 53 | 45 |
| **53** | 72 | 70 | 54 | 47 |
| **54** | 73 | 71 | 56 | 48 |
| **55** | 74 | 73 | 57 | 50 |
| **56** | 76 | 74 | 59 | 51 |
| **57** | 77 | 75 | 60 | 53 |
| **58** | 78 | 76 | 62 | 54 |
| **59** | 80 | 77 | 63 | 56 |
| **60** | 81 | 78 | 65 | 57 |
| **61** | 82 | 80 | 66 | 59 |
| **62** | 83 | 81 | 67 | 60 |
| **63** | 85 | 82 | 69 | 62 |
| **64** | 86 | 83 | 70 | 63 |
| **65** | 87 | 84 | 72 | 65 |
| **66** | 88 | 86 | 73 | 66 |
| **67** | 90 | 87 | 75 | 67 |
| **68** | 91 | 88 | 76 | 69 |
| **69** | 92 | 89 | 78 | 70 |
| **70** | 94 | 90 | 79 | 72 |
| **71** | 95 | 91 | 81 | 73 |
| **72** | 96 | 93 | 82 | 75 |
| **73** | 97 | 94 | 84 | 76 |
| **74** | 99 | 95 | 85 | 78 |
| **75** | 100 | 96 | 87 | 79 |
| **76** | 101 | 97 | 88 | 81 |
| **77** | 102 | 99 | 90 | 82 |
| **78** | 104 | 100 | 91 | 84 |
| **79** | 105 | 101 | 93 | 85 |
| **80** | 106 | 102 | 94 | 87 |
| **81** | 108 | 103 | 96 | 88 |
| **82** | 109 | 104 | 97 | 90 |
| **83** | 110 | 106 | 99 | 91 |
| **84** | 111 | 107 | 100 | 93 |
| **85** | 113 | 108 | 102 | 94 |
| **86** | 114 | 109 | 103 | 95 |
| **87** | 115 | 110 | 104 | 97 |
| **88** | 117 | 111 | 106 | 98 |
| **89** | 118 | 113 | 107 | 100 |
| **90** | 119 | 114 | 109 | 101 |
| **91** | 120 | 115 | 110 | 103 |
| **92** | 122 | 116 | 112 | 104 |
| **93** | 123 | 117 | 113 | 106 |
| **94** | 124 | 119 | 115 | 107 |
| **95** | 125 | 120 | 116 | 109 |
| **96** | 127 | 121 | 118 | 110 |
| **97** | 128 | 122 | 119 | 112 |
| **98** | 129 | 123 | 121 | 113 |
| **99** | 131 | 124 | 122 | 115 |
| **100** | 132 | 126 | 124 | 116 |
| **101** | 133 | 127 | 125 | 118 |
| **102** | 134 | 128 | 127 | 119 |
| **103** | 136 | 129 | 128 | 121 |
| **104** | 137 | 130 | 130 | 122 |
| **105** | 138 | 132 | 131 | 123 |
| **106** | 139 | 133 | 133 | 125 |
| **107** | 141 | 134 | 134 | 126 |
| **108** | 142 | 135 | 136 | 128 |
| **109** | 143 | 136 | 137 | 129 |
| **110** | 145 | 137 | 139 | 131 |
| **111** | 146 | 139 | 140 | 132 |
| **112** | 147 | 140 | 141 | 134 |
| **113** | 148 | 141 | 143 | 135 |
| **114** | 150 | 142 | 144 | 137 |
| **115** |  | 143 | 146 | 138 |
| **116** |  | 145 | 147 | 140 |
| **117** |  | 146 | 149 | 141 |
| **118** |  | 147 | 150 | 143 |
| **119** |  | 148 | 148 | 144 |
| **120** |  | 149 | 149 | 146 |
| **121** |  | 150 | 151 | 147 |
| **122** |  |  |  | 149 |
| **123** |  |  |  | 150 |
| **124** |  |  |  | 151 |
| **125** |  |  |  | 137 |
| **126** |  |  |  | 139 |
| **127** |  |  |  | 140 |
| **128** |  |  |  | 141 |
| **129** |  |  |  | 143 |
| **130** |  |  |  | 144 |
| **131** |  |  |  | 146 |
| **132** |  |  |  | 147 |
| **133** |  |  |  | 148 |
| **134** |  |  |  | 150 |

| **Table C2.** Raw scores secondary school 'Moving Sideways' (JS) (Girls) | | | | |
| --- | --- | --- | --- | --- |
| **Raw Score / Age (years)** | **12-13.99** | **14-15.99** | **16-17.99** | **18-19.99** |
| **1** | 12 | 16 | 3 | 0 |
| **2** | 14 | 17 | 5 | 0 |
| **3** | 15 | 19 | 6 | 0 |
| **4** | 17 | 20 | 8 | 0 |
| **5** | 19 | 22 | 10 | 2 |
| **6** | 20 | 23 | 11 | 4 |
| **7** | 22 | 25 | 13 | 5 |
| **8** | 24 | 26 | 15 | 7 |
| **9** | 26 | 28 | 16 | 8 |
| **10** | 27 | 29 | 18 | 10 |
| **11** | 29 | 31 | 20 | 11 |
| **12** | 31 | 32 | 21 | 13 |
| **13** | 33 | 34 | 23 | 15 |
| **14** | 34 | 36 | 25 | 16 |
| **15** | 36 | 37 | 26 | 18 |
| **16** | 38 | 39 | 28 | 19 |
| **17** | 39 | 40 | 30 | 21 |
| **18** | 41 | 42 | 31 | 23 |
| **19** | 43 | 43 | 33 | 24 |
| **20** | 45 | 45 | 35 | 26 |
| **21** | 46 | 46 | 36 | 27 |
| **22** | 48 | 48 | 38 | 29 |
| **23** | 50 | 49 | 40 | 30 |
| **24** | 51 | 51 | 41 | 32 |
| **25** | 53 | 52 | 43 | 34 |
| **26** | 55 | 54 | 45 | 35 |
| **27** | 57 | 55 | 46 | 37 |
| **28** | 58 | 57 | 48 | 38 |
| **29** | 60 | 58 | 50 | 40 |
| **30** | 62 | 60 | 51 | 42 |
| **31** | 64 | 61 | 53 | 43 |
| **32** | 65 | 63 | 55 | 45 |
| **33** | 67 | 64 | 56 | 46 |
| **34** | 69 | 66 | 58 | 48 |
| **35** | 70 | 67 | 60 | 49 |
| **36** | 72 | 69 | 61 | 51 |
| **37** | 74 | 71 | 63 | 53 |
| **38** | 76 | 72 | 65 | 54 |
| **39** | 77 | 74 | 66 | 56 |
| **40** | 79 | 75 | 68 | 57 |
| **41** | 81 | 77 | 70 | 59 |
| **42** | 82 | 78 | 71 | 61 |
| **43** | 84 | 80 | 73 | 62 |
| **44** | 86 | 81 | 75 | 64 |
| **45** | 88 | 83 | 76 | 65 |
| **46** | 89 | 84 | 78 | 67 |
| **47** | 91 | 86 | 80 | 68 |
| **48** | 93 | 87 | 81 | 70 |
| **49** | 94 | 89 | 83 | 72 |
| **50** | 96 | 90 | 85 | 73 |
| **51** | 98 | 92 | 86 | 75 |
| **52** | 100 | 93 | 88 | 76 |
| **53** | 101 | 95 | 90 | 78 |
| **54** | 103 | 96 | 91 | 80 |
| **55** | 105 | 98 | 93 | 81 |
| **56** | 107 | 99 | 95 | 83 |
| **57** | 108 | 101 | 96 | 84 |
| **58** | 110 | 102 | 98 | 86 |
| **59** | 112 | 104 | 100 | 88 |
| **60** | 113 | 105 | 101 | 89 |
| **61** | 115 | 107 | 103 | 91 |
| **62** | 117 | 109 | 105 | 92 |
| **63** | 119 | 110 | 106 | 94 |
| **64** | 120 | 112 | 108 | 95 |
| **65** | 122 | 113 | 110 | 97 |
| **66** | 124 | 115 | 111 | 99 |
| **67** | 125 | 116 | 113 | 100 |
| **68** | 127 | 118 | 115 | 102 |
| **69** | 129 | 119 | 116 | 103 |
| **70** | 131 | 121 | 118 | 105 |
| **71** | 132 | 122 | 120 | 107 |
| **72** | 134 | 124 | 121 | 108 |
| **73** | 136 | 125 | 123 | 110 |
| **74** | 138 | 127 | 125 | 111 |
| **75** | 139 | 128 | 126 | 113 |
| **76** | 141 | 130 | 128 | 114 |
| **77** | 143 | 131 | 130 | 116 |
| **78** | 144 | 133 | 131 | 118 |
| **79** | 146 | 134 | 133 | 119 |
| **80** | 148 | 136 | 135 | 121 |
| **81** | 150 | 137 | 136 | 122 |
| **82** |  | 139 | 138 | 124 |
| **83** |  | 140 | 140 | 126 |
| **84** |  | 142 | 141 | 127 |
| **85** |  | 144 | 143 | 129 |
| **86** |  | 145 | 145 | 130 |
| **87** |  | 147 | 146 | 132 |
| **88** |  | 148 | 148 | 133 |
| **89** |  | 150 | 150 | 135 |
| **90** |  |  |  | 137 |
| **91** |  |  |  | 138 |
| **92** |  |  |  | 140 |
| **93** |  |  |  | 141 |
| **94** |  |  |  | 143 |
| **95** |  |  |  | 145 |
| **96** |  |  |  | 146 |
| **97** |  |  |  | 148 |
| **98** |  |  |  | 149 |
| **99** |  |  |  | 151 |

| **Table C3.** Raw scores secondary school ‘Balancing Backwards’ (BB) (Girls) | | | | | |
| --- | --- | --- | --- | --- | --- |
| **Raw Score / Age (years)** | **12-13.99** | **14-15.99** | **16-17.99** | **18-19.99** |  |
| **1** | 38 | 32 | 10 | 26 |  |
| **2** | 39 | 33 | 12 | 27 |  |
| **3** | 40 | 35 | 13 | 29 |  |
| **4** | 41 | 36 | 15 | 30 |  |
| **5** | 43 | 37 | 16 | 31 |  |
| **6** | 44 | 38 | 18 | 33 |  |
| **7** | 45 | 40 | 20 | 34 |  |
| **8** | 46 | 41 | 21 | 36 |  |
| **9** | 47 | 42 | 23 | 37 |  |
| **10** | 48 | 44 | 24 | 38 |  |
| **11** | 50 | 45 | 26 | 40 |  |
| **12** | 51 | 46 | 28 | 41 |  |
| **13** | 52 | 47 | 29 | 42 |  |
| **14** | 53 | 49 | 31 | 44 |  |
| **15** | 54 | 50 | 32 | 45 |  |
| **16** | 55 | 51 | 34 | 46 |  |
| **17** | 57 | 52 | 36 | 48 |  |
| **18** | 58 | 54 | 37 | 49 |  |
| **19** | 59 | 55 | 39 | 50 |  |
| **20** | 60 | 56 | 40 | 52 |  |
| **21** | 61 | 58 | 42 | 53 |  |
| **22** | 62 | 59 | 44 | 54 |  |
| **23** | 64 | 60 | 45 | 56 |  |
| **24** | 65 | 61 | 47 | 57 |  |
| **25** | 66 | 63 | 48 | 58 |  |
| **26** | 67 | 64 | 50 | 60 |  |
| **27** | 68 | 65 | 52 | 61 |  |
| **28** | 70 | 66 | 53 | 62 |  |
| **29** | 71 | 68 | 55 | 64 |  |
| **30** | 72 | 69 | 56 | 65 |  |
| **31** | 73 | 70 | 58 | 66 |  |
| **32** | 74 | 72 | 60 | 68 |  |
| **33** | 75 | 73 | 61 | 69 |  |
| **34** | 77 | 74 | 63 | 70 |  |
| **35** | 78 | 75 | 64 | 72 |  |
| **36** | 79 | 77 | 66 | 73 |  |
| **37** | 80 | 78 | 68 | 74 |  |
| **38** | 81 | 79 | 69 | 76 |  |
| **39** | 82 | 80 | 71 | 77 |  |
| **40** | 84 | 82 | 72 | 78 |  |
| **41** | 85 | 83 | 74 | 80 |  |
| **42** | 86 | 84 | 76 | 81 |  |
| **43** | 87 | 86 | 77 | 82 |  |
| **44** | 88 | 87 | 79 | 84 |  |
| **45** | 89 | 88 | 80 | 85 |  |
| **46** | 91 | 89 | 82 | 86 |  |
| **47** | 92 | 91 | 84 | 88 |  |
| **48** | 93 | 92 | 85 | 89 |  |
| **49** | 94 | 93 | 87 | 90 |  |
| **50** | 95 | 94 | 88 | 92 |  |
| **51** | 96 | 96 | 90 | 93 |  |
| **52** | 98 | 97 | 92 | 94 |  |
| **53** | 99 | 98 | 93 | 96 |  |
| **54** | 100 | 100 | 95 | 97 |  |
| **55** | 101 | 101 | 96 | 98 |  |
| **56** | 102 | 102 | 98 | 100 |  |
| **57** | 104 | 103 | 100 | 101 |  |
| **58** | 105 | 105 | 101 | 102 |  |
| **59** | 106 | 106 | 103 | 104 |  |
| **60** | 107 | 107 | 104 | 105 |  |
| **61** | 108 | 109 | 106 | 106 |  |
| **62** | 109 | 110 | 108 | 108 |  |
| **63** | 111 | 111 | 109 | 109 |  |
| **64** | 112 | 112 | 111 | 110 |  |
| **65** | 113 | 114 | 112 | 112 |  |
| **66** | 114 | 115 | 114 | 113 |  |
| **67** | 115 | 116 | 116 | 114 |  |
| **68** | 116 | 117 | 117 | 116 |  |
| **69** | 118 | 119 | 119 | 117 |  |
| **70** | 119 | 120 | 120 | 118 |  |
| **71** | 120 | 121 | 122 | 120 |  |
| **72** | 121 | 123 | 124 | 121 |  |

| **Table C4.** Raw scores secondary school 'Eye-Hand Coordination' (EHC) (Girls) | | | | |
| --- | --- | --- | --- | --- |
| **Raw Score / Age (years)** | **12-13.99** | **14-15.99** | **16-17.99** | **18-19.99** |
| **0** | 67 | 78 | 47 | 39 |
| **1** | 68 | 78 | 48 | 40 |
| **2** | 69 | 79 | 49 | 41 |
| **3** | 70 | 80 | 51 | 42 |
| **4** | 71 | 80 | 52 | 43 |
| **5** | 72 | 81 | 53 | 44 |
| **6** | 73 | 82 | 54 | 45 |
| **7** | 74 | 83 | 55 | 46 |
| **8** | 75 | 83 | 56 | 47 |
| **9** | 76 | 84 | 57 | 48 |
| **10** | 77 | 85 | 58 | 49 |
| **11** | 79 | 85 | 60 | 50 |
| **12** | 80 | 86 | 61 | 51 |
| **13** | 81 | 87 | 62 | 52 |
| **14** | 82 | 88 | 63 | 53 |
| **15** | 83 | 88 | 64 | 54 |
| **16** | 84 | 89 | 65 | 55 |
| **17** | 85 | 90 | 66 | 56 |
| **18** | 86 | 90 | 68 | 57 |
| **19** | 87 | 91 | 69 | 58 |
| **20** | 88 | 92 | 70 | 59 |
| **21** | 89 | 93 | 71 | 60 |
| **22** | 90 | 93 | 72 | 61 |
| **23** | 91 | 94 | 73 | 62 |
| **24** | 92 | 95 | 74 | 63 |
| **25** | 93 | 95 | 75 | 64 |
| **26** | 94 | 96 | 77 | 65 |
| **27** | 95 | 97 | 78 | 66 |
| **28** | 96 | 98 | 79 | 67 |
| **29** | 97 | 98 | 80 | 68 |
| **30** | 98 | 99 | 81 | 69 |
| **31** | 99 | 100 | 82 | 70 |
| **32** | 100 | 100 | 83 | 71 |
| **33** | 101 | 101 | 84 | 72 |
| **34** | 102 | 102 | 86 | 73 |
| **35** | 103 | 103 | 87 | 74 |
| **36** | 104 | 103 | 88 | 75 |
| **37** | 105 | 104 | 89 | 76 |
| **38** | 106 | 105 | 90 | 77 |
| **39** | 107 | 105 | 91 | 78 |
| **40** | 108 | 106 | 92 | 79 |
| **41** | 109 | 107 | 93 | 80 |
| **42** | 110 | 108 | 95 | 81 |
| **43** | 111 | 108 | 96 | 82 |
| **44** | 112 | 109 | 97 | 83 |
| **45** | 113 | 110 | 98 | 84 |
| **46** | 114 | 110 | 99 | 85 |
| **47** | 115 | 111 | 100 | 86 |
| **48** | 116 | 112 | 101 | 87 |
| **49** | 117 | 113 | 103 | 88 |
| **50** | 118 | 113 | 104 | 89 |
| **51** | 119 | 114 | 105 | 90 |
| **52** | 120 | 115 | 106 | 91 |
| **53** | 121 | 115 | 107 | 92 |
| **54** | 122 | 116 | 108 | 93 |
| **55** | 123 | 117 | 109 | 94 |
| **56** | 124 | 118 | 110 | 95 |
| **57** | 125 | 118 | 112 | 96 |
| **58** | 126 | 119 | 113 | 97 |
| **59** | 127 | 120 | 114 | 98 |
| **60** | 128 | 120 | 115 | 99 |
| **61** | 129 | 121 | 116 | 100 |
| **62** | 130 | 122 | 117 | 101 |
| **63** | 131 | 123 | 118 | 102 |
| **64** | 132 | 123 | 119 | 103 |
| **65** | 133 | 124 | 121 | 104 |
| **66** | 134 | 125 | 122 | 105 |
| **67** | 135 | 125 | 123 | 106 |
| **68** | 136 | 126 | 124 | 107 |
| **69** | 137 | 127 | 125 | 108 |
| **70** | 138 | 128 | 126 | 109 |
| **71** | 139 | 128 | 127 | 110 |
| **72** | 140 | 129 | 128 | 111 |
| **73** | 141 | 130 | 130 | 112 |
| **74** | 142 | 130 | 131 | 113 |
| **75** | 143 | 131 | 132 | 114 |
| **76** | 144 | 132 | 133 | 115 |
| **77** | 145 | 133 | 134 | 116 |
| **78** | 146 | 133 | 135 | 117 |
| **79** | 147 | 134 | 136 | 118 |
| **80** | 148 | 135 | 138 | 119 |
| **81** | 149 | 135 | 139 | 120 |
| **82** | 150 | 136 | 140 | 121 |
| **83** |  | 137 | 141 | 122 |
| **84** |  | 138 | 142 | 123 |
| **85** |  | 138 | 143 | 124 |
| **86** |  | 139 | 144 | 125 |
| **87** |  | 140 | 145 | 126 |
| **88** |  | 140 | 147 | 127 |
| **89** |  | 141 | 148 | 128 |
| **90** |  | 142 | 149 | 129 |
| **91** |  | 143 | 150 | 129 |
| **92** |  | 143 |  | 130 |
| **93** |  | 144 |  | 131 |
| **94** |  | 145 |  | 132 |
| **95** |  | 145 |  | 133 |
| **96** |  | 146 |  | 134 |
| **97** |  | 147 |  | 135 |
| **98** |  | 148 |  | 136 |
| **99** |  | 148 |  | 137 |
| **100** |  | 149 |  | 138 |
| **101** |  | 150 |  | 139 |
| **102** |  |  |  | 140 |
| **103** |  |  |  | 141 |
| **104** |  |  |  | 142 |
| **105** |  |  |  | 143 |
| **106** |  |  |  | 144 |
| **107** |  |  |  | 145 |
| **108** |  |  |  | 146 |
| **109** |  |  |  | 147 |
| **110** |  |  |  | 148 |
| **111** |  |  |  | 149 |
| **112** |  |  |  | 150 |

| **Table C5.** Raw scores secondary school 'Jumping Sideways' (JS) (Boys) | | | | |
| --- | --- | --- | --- | --- |
| **Raw Score / Age (years)** | **12-13.99** | **14-15.99** | **16-17.99** | **18-19.99** |
| **1** | 0 | 2 | 1 | 0 |
| **2** | 0 | 3 | 2 | 0 |
| **3** | 1 | 4 | 3 | 0 |
| **4** | 3 | 5 | 4 | 0 |
| **5** | 4 | 6 | 5 | 0 |
| **6** | 5 | 8 | 6 | 0 |
| **7** | 6 | 9 | 7 | 0 |
| **8** | 8 | 10 | 8 | 0 |
| **9** | 9 | 11 | 10 | 0 |
| **10** | 10 | 12 | 11 | 0 |
| **11** | 12 | 14 | 12 | 0 |
| **12** | 13 | 15 | 13 | 0 |
| **13** | 14 | 16 | 14 | 0 |
| **14** | 15 | 17 | 15 | 0 |
| **15** | 17 | 18 | 16 | 0 |
| **16** | 18 | 20 | 17 | 0 |
| **17** | 19 | 21 | 18 | 0 |
| **18** | 21 | 22 | 19 | 0 |
| **19** | 22 | 23 | 21 | 0 |
| **20** | 23 | 24 | 22 | 0 |
| **21** | 24 | 25 | 23 | 0 |
| **22** | 26 | 27 | 24 | 1 |
| **23** | 27 | 28 | 25 | 3 |
| **24** | 28 | 29 | 26 | 4 |
| **25** | 30 | 30 | 27 | 5 |
| **26** | 31 | 31 | 28 | 7 |
| **27** | 32 | 33 | 29 | 8 |
| **28** | 33 | 34 | 30 | 9 |
| **29** | 35 | 35 | 32 | 11 |
| **30** | 36 | 36 | 33 | 12 |
| **31** | 37 | 37 | 34 | 13 |
| **32** | 38 | 39 | 35 | 14 |
| **33** | 40 | 40 | 36 | 16 |
| **34** | 41 | 41 | 37 | 17 |
| **35** | 42 | 42 | 38 | 18 |
| **36** | 44 | 43 | 39 | 20 |
| **37** | 45 | 45 | 40 | 21 |
| **38** | 46 | 46 | 41 | 22 |
| **39** | 47 | 47 | 43 | 24 |
| **40** | 49 | 48 | 44 | 25 |
| **41** | 50 | 49 | 45 | 26 |
| **42** | 51 | 50 | 46 | 27 |
| **43** | 53 | 52 | 47 | 29 |
| **44** | 54 | 53 | 48 | 30 |
| **45** | 55 | 54 | 49 | 31 |
| **46** | 56 | 55 | 50 | 33 |
| **47** | 58 | 56 | 51 | 34 |
| **48** | 59 | 58 | 52 | 35 |
| **49** | 60 | 59 | 54 | 37 |
| **50** | 62 | 60 | 55 | 38 |
| **51** | 63 | 61 | 56 | 39 |
| **52** | 64 | 62 | 57 | 40 |
| **53** | 65 | 64 | 58 | 42 |
| **54** | 67 | 65 | 59 | 43 |
| **55** | 68 | 66 | 60 | 44 |
| **56** | 69 | 67 | 61 | 46 |
| **57** | 71 | 68 | 62 | 47 |
| **58** | 72 | 70 | 63 | 48 |
| **59** | 73 | 71 | 64 | 50 |
| **60** | 74 | 72 | 66 | 51 |
| **61** | 76 | 73 | 67 | 52 |
| **62** | 77 | 74 | 68 | 53 |
| **63** | 78 | 75 | 69 | 55 |
| **64** | 79 | 77 | 70 | 56 |
| **65** | 81 | 78 | 71 | 57 |
| **66** | 82 | 79 | 72 | 59 |
| **67** | 83 | 80 | 73 | 60 |
| **68** | 85 | 81 | 74 | 61 |
| **69** | 86 | 83 | 75 | 63 |
| **70** | 87 | 84 | 77 | 64 |
| **71** | 88 | 85 | 78 | 65 |
| **72** | 90 | 86 | 79 | 66 |
| **73** | 91 | 87 | 80 | 68 |
| **74** | 92 | 89 | 81 | 69 |
| **75** | 94 | 90 | 82 | 70 |
| **76** | 95 | 91 | 83 | 72 |
| **77** | 96 | 92 | 84 | 73 |
| **78** | 97 | 93 | 85 | 74 |
| **79** | 99 | 95 | 86 | 76 |
| **80** | 100 | 96 | 88 | 77 |
| **81** | 101 | 97 | 89 | 78 |
| **82** | 103 | 98 | 90 | 79 |
| **83** | 104 | 99 | 91 | 81 |
| **84** | 105 | 100 | 92 | 82 |
| **85** | 106 | 102 | 93 | 83 |
| **86** | 108 | 103 | 94 | 85 |
| **87** | 109 | 104 | 95 | 86 |
| **88** | 110 | 105 | 96 | 87 |
| **89** | 111 | 106 | 97 | 89 |
| **90** | 113 | 108 | 99 | 90 |
| **91** | 114 | 109 | 100 | 91 |
| **92** | 115 | 110 | 101 | 92 |
| **93** | 117 | 111 | 102 | 94 |
| **94** | 118 | 112 | 103 | 95 |
| **95** | 119 | 114 | 104 | 96 |
| **96** | 120 | 115 | 105 | 98 |
| **97** | 122 | 116 | 106 | 99 |
| **98** | 123 | 117 | 107 | 100 |
| **99** | 124 | 118 | 108 | 102 |
| **100** | 126 | 119 | 110 | 103 |
| **101** | 127 | 121 | 111 | 104 |
| **102** | 128 | 122 | 112 | 105 |
| **103** | 129 | 123 | 113 | 107 |
| **104** | 131 | 124 | 114 | 108 |
| **105** | 132 | 125 | 115 | 109 |
| **106** | 133 | 127 | 116 | 111 |
| **107** | 135 | 128 | 117 | 112 |
| **108** | 136 | 129 | 118 | 113 |
| **109** | 137 | 130 | 119 | 115 |
| **110** | 138 | 131 | 121 | 116 |
| **111** | 140 | 133 | 122 | 117 |
| **112** | 141 | 134 | 123 | 118 |
| **113** | 142 | 135 | 124 | 120 |
| **114** | 144 | 136 | 125 | 121 |
| **115** | 145 | 137 | 126 | 122 |
| **116** | 146 | 139 | 127 | 124 |
| **117** | 147 | 140 | 128 | 125 |
| **118** | 149 | 141 | 129 | 126 |
| **119** | 150 | 142 | 130 | 128 |
| **120** |  | 143 | 131 | 129 |
| **121** |  | 144 | 133 | 130 |
| **122** |  | 146 | 134 | 131 |
| **123** |  | 147 | 135 | 133 |
| **124** |  | 148 | 136 | 134 |
| **125** |  | 149 | 137 | 135 |
| **126** |  | 150 | 138 | 137 |
| **127** |  |  | 139 | 138 |
| **128** |  |  | 140 | 139 |
| **129** |  |  | 141 | 141 |
| **130** |  |  | 142 | 142 |
| **131** |  |  | 144 | 143 |
| **132** |  |  | 145 | 144 |
| **133** |  |  | 146 | 146 |
| **134** |  |  | 147 | 147 |
| **135** |  |  | 148 | 148 |
| **136** |  |  | 149 | 150 |
| **137** |  |  | 150 |  |

| **Table C6.** Raw scores secondary school 'Moving Sideways' (JS) (Boys) | | | | |
| --- | --- | --- | --- | --- |
| **Raw Score / Age (years)** | **12-13.99** | **14-15.99** | **16-17.99** | **18-19.99** |
| **1** | 0 | 23 | 9 | 9 |
| **2** | 0 | 24 | 11 | 11 |
| **3** | 2 | 25 | 12 | 12 |
| **4** | 4 | 27 | 14 | 13 |
| **5** | 6 | 28 | 15 | 15 |
| **6** | 8 | 30 | 16 | 16 |
| **7** | 10 | 31 | 18 | 17 |
| **8** | 12 | 32 | 19 | 19 |
| **9** | 14 | 34 | 21 | 20 |
| **10** | 16 | 35 | 22 | 21 |
| **11** | 18 | 37 | 24 | 23 |
| **12** | 20 | 38 | 25 | 24 |
| **13** | 22 | 39 | 27 | 25 |
| **14** | 24 | 41 | 28 | 27 |
| **15** | 26 | 42 | 30 | 28 |
| **16** | 28 | 44 | 31 | 29 |
| **17** | 30 | 45 | 33 | 31 |
| **18** | 32 | 47 | 34 | 32 |
| **19** | 34 | 48 | 36 | 33 |
| **20** | 36 | 49 | 37 | 35 |
| **21** | 37 | 51 | 39 | 36 |
| **22** | 39 | 52 | 40 | 37 |
| **23** | 41 | 54 | 41 | 39 |
| **24** | 43 | 55 | 43 | 40 |
| **25** | 45 | 56 | 44 | 41 |
| **26** | 47 | 58 | 46 | 43 |
| **27** | 49 | 59 | 47 | 44 |
| **28** | 51 | 61 | 49 | 45 |
| **29** | 53 | 62 | 50 | 47 |
| **30** | 55 | 63 | 52 | 48 |
| **31** | 57 | 65 | 53 | 49 |
| **32** | 59 | 66 | 55 | 51 |
| **33** | 61 | 68 | 56 | 52 |
| **34** | 63 | 69 | 58 | 53 |
| **35** | 65 | 70 | 59 | 55 |
| **36** | 67 | 72 | 61 | 56 |
| **37** | 69 | 73 | 62 | 57 |
| **38** | 71 | 75 | 64 | 59 |
| **39** | 73 | 76 | 65 | 60 |
| **40** | 75 | 77 | 66 | 61 |
| **41** | 76 | 79 | 68 | 63 |
| **42** | 78 | 80 | 69 | 64 |
| **43** | 80 | 82 | 71 | 65 |
| **44** | 82 | 83 | 72 | 67 |
| **45** | 84 | 84 | 74 | 68 |
| **46** | 86 | 86 | 75 | 69 |
| **47** | 88 | 87 | 77 | 71 |
| **48** | 90 | 89 | 78 | 72 |
| **49** | 92 | 90 | 80 | 73 |
| **50** | 94 | 92 | 81 | 75 |
| **51** | 96 | 93 | 83 | 76 |
| **52** | 98 | 94 | 84 | 77 |
| **53** | 100 | 96 | 86 | 79 |
| **54** | 102 | 97 | 87 | 80 |
| **55** | 104 | 99 | 89 | 81 |
| **56** | 106 | 100 | 90 | 83 |
| **57** | 108 | 101 | 91 | 84 |
| **58** | 110 | 103 | 93 | 85 |
| **59** | 112 | 104 | 94 | 87 |
| **60** | 113 | 106 | 96 | 88 |
| **61** | 115 | 107 | 97 | 89 |
| **62** | 117 | 108 | 99 | 91 |
| **63** | 119 | 110 | 100 | 92 |
| **64** | 121 | 111 | 102 | 93 |
| **65** | 123 | 113 | 103 | 95 |
| **66** | 125 | 114 | 105 | 96 |
| **67** | 127 | 115 | 106 | 97 |
| **68** | 129 | 117 | 108 | 99 |
| **69** | 131 | 118 | 109 | 100 |
| **70** | 133 | 120 | 111 | 101 |
| **71** | 135 | 121 | 112 | 103 |
| **72** | 137 | 122 | 113 | 104 |
| **73** | 139 | 124 | 115 | 105 |
| **74** | 141 | 125 | 116 | 107 |
| **75** | 143 | 127 | 118 | 108 |
| **76** | 145 | 128 | 119 | 109 |
| **77** | 147 | 129 | 121 | 111 |
| **78** | 149 | 131 | 122 | 112 |
| **79** | 151 | 132 | 124 | 113 |
| **80** | 150 | 134 | 125 | 115 |
| **81** |  | 135 | 127 | 116 |
| **82** |  | 137 | 128 | 117 |
| **83** |  | 138 | 130 | 119 |
| **84** |  | 139 | 131 | 120 |
| **85** |  | 141 | 133 | 121 |
| **86** |  | 142 | 134 | 123 |
| **87** |  | 144 | 136 | 124 |
| **88** |  | 145 | 137 | 125 |
| **89** |  | 146 | 138 | 127 |
| **90** |  | 148 | 140 | 128 |
| **91** |  | 149 | 141 | 129 |
| **92** |  | 151 | 143 | 131 |
| **93** |  |  | 144 | 132 |
| **94** |  |  | 146 | 133 |
| **95** |  |  | 147 | 135 |
| **96** |  |  | 149 | 136 |
| **97** |  |  | 150 | 137 |
| **98** |  |  |  | 139 |
| **99** |  |  |  | 140 |
| **100** |  |  |  | 141 |
| **101** |  |  |  | 143 |
| **102** |  |  |  | 144 |
| **103** |  |  |  | 145 |
| **104** |  |  |  | 147 |
| **105** |  |  |  | 148 |
| **106** |  |  |  | 149 |
| **107** |  |  |  | 151 |

| **Table C7.** Raw scores secondary school 'Balancing Backwards' (BB) (Boys) | | | | | |
| --- | --- | --- | --- | --- | --- |
| **Raw Score / Age (years)** | **12-13.99** | **14-15.99** | **16-17.99** | **18-19.99** |  |
| **1** | 38 | 42 | 37 | 50 |  |
| **2** | 40 | 43 | 38 | 51 |  |
| **3** | 41 | 44 | 40 | 52 |  |
| **4** | 42 | 45 | 41 | 53 |  |
| **5** | 43 | 46 | 42 | 54 |  |
| **6** | 45 | 47 | 43 | 55 |  |
| **7** | 46 | 49 | 44 | 56 |  |
| **8** | 47 | 50 | 46 | 57 |  |
| **9** | 48 | 51 | 47 | 58 |  |
| **10** | 49 | 52 | 48 | 59 |  |
| **11** | 51 | 53 | 49 | 60 |  |
| **12** | 52 | 54 | 50 | 61 |  |
| **13** | 53 | 56 | 52 | 62 |  |
| **14** | 54 | 57 | 53 | 63 |  |
| **15** | 56 | 58 | 54 | 64 |  |
| **16** | 57 | 59 | 55 | 64 |  |
| **17** | 58 | 60 | 56 | 65 |  |
| **18** | 59 | 61 | 57 | 66 |  |
| **19** | 61 | 63 | 59 | 67 |  |
| **20** | 62 | 64 | 60 | 68 |  |
| **21** | 63 | 65 | 61 | 69 |  |
| **22** | 64 | 66 | 62 | 70 |  |
| **23** | 66 | 67 | 63 | 71 |  |
| **24** | 67 | 68 | 65 | 72 |  |
| **25** | 68 | 70 | 66 | 73 |  |
| **26** | 69 | 71 | 67 | 74 |  |
| **27** | 71 | 72 | 68 | 75 |  |
| **28** | 72 | 73 | 69 | 76 |  |
| **29** | 73 | 74 | 70 | 77 |  |
| **30** | 74 | 75 | 72 | 78 |  |
| **31** | 75 | 77 | 73 | 79 |  |
| **32** | 77 | 78 | 74 | 79 |  |
| **33** | 78 | 79 | 75 | 80 |  |
| **34** | 79 | 80 | 76 | 81 |  |
| **35** | 80 | 81 | 78 | 82 |  |
| **36** | 82 | 82 | 79 | 83 |  |
| **37** | 83 | 84 | 80 | 84 |  |
| **38** | 84 | 85 | 81 | 85 |  |
| **39** | 85 | 86 | 82 | 86 |  |
| **40** | 87 | 87 | 84 | 87 |  |
| **41** | 88 | 88 | 85 | 88 |  |
| **42** | 89 | 90 | 86 | 89 |  |
| **43** | 90 | 91 | 87 | 90 |  |
| **44** | 92 | 92 | 88 | 91 |  |
| **45** | 93 | 93 | 89 | 92 |  |
| **46** | 94 | 94 | 91 | 93 |  |
| **47** | 95 | 95 | 92 | 94 |  |
| **48** | 97 | 97 | 93 | 94 |  |
| **49** | 98 | 98 | 94 | 95 |  |
| **50** | 99 | 99 | 95 | 96 |  |
| **51** | 100 | 100 | 97 | 97 |  |
| **52** | 101 | 101 | 98 | 98 |  |
| **53** | 103 | 102 | 99 | 99 |  |
| **54** | 104 | 104 | 100 | 100 |  |
| **55** | 105 | 105 | 101 | 101 |  |
| **56** | 106 | 106 | 103 | 102 |  |
| **57** | 108 | 107 | 104 | 103 |  |
| **58** | 109 | 108 | 105 | 104 |  |
| **59** | 110 | 109 | 106 | 105 |  |
| **60** | 111 | 111 | 107 | 106 |  |
| **61** | 113 | 112 | 108 | 107 |  |
| **62** | 114 | 113 | 110 | 108 |  |
| **63** | 115 | 114 | 111 | 109 |  |
| **64** | 116 | 115 | 112 | 109 |  |
| **65** | 118 | 116 | 113 | 110 |  |
| **66** | 119 | 118 | 114 | 111 |  |
| **67** | 120 | 119 | 116 | 112 |  |
| **68** | 121 | 120 | 117 | 113 |  |
| **69** | 123 | 121 | 118 | 114 |  |
| **70** | 124 | 122 | 119 | 115 |  |
| **71** | 125 | 123 | 120 | 116 |  |
| **72** | 126 | 125 | 121 | 117 |  |

| **Table C8.** Raw scores secondary school 'Eye-Hand Coordination' (EHC) (Boys) | | | | | |
| --- | --- | --- | --- | --- | --- |
| **Raw Score / Age (years)** | **12-13.99** | **14-15.99** | **16-17.99** | **18-19.99** |  |
| **0** | 51 | 73 | 41 | 30 |  |
| **1** | 52 | 74 | 42 | 31 |  |
| **2** | 53 | 74 | 43 | 32 |  |
| **3** | 54 | 75 | 44 | 33 |  |
| **4** | 55 | 76 | 45 | 34 |  |
| **5** | 56 | 76 | 46 | 35 |  |
| **6** | 57 | 77 | 47 | 37 |  |
| **7** | 58 | 77 | 48 | 38 |  |
| **8** | 59 | 78 | 49 | 39 |  |
| **9** | 60 | 79 | 50 | 40 |  |
| **10** | 61 | 79 | 51 | 41 |  |
| **11** | 62 | 80 | 52 | 42 |  |
| **12** | 63 | 80 | 53 | 43 |  |
| **13** | 64 | 81 | 53 | 44 |  |
| **14** | 65 | 82 | 54 | 45 |  |
| **15** | 67 | 82 | 55 | 46 |  |
| **16** | 68 | 83 | 56 | 47 |  |
| **17** | 69 | 83 | 57 | 48 |  |
| **18** | 70 | 84 | 58 | 49 |  |
| **19** | 71 | 85 | 59 | 50 |  |
| **20** | 72 | 85 | 60 | 51 |  |
| **21** | 73 | 86 | 61 | 52 |  |
| **22** | 74 | 87 | 62 | 53 |  |
| **23** | 75 | 87 | 63 | 54 |  |
| **24** | 76 | 88 | 64 | 55 |  |
| **25** | 77 | 88 | 65 | 56 |  |
| **26** | 78 | 89 | 66 | 57 |  |
| **27** | 79 | 90 | 67 | 58 |  |
| **28** | 80 | 90 | 68 | 59 |  |
| **29** | 81 | 91 | 69 | 60 |  |
| **30** | 82 | 91 | 70 | 61 |  |
| **31** | 83 | 92 | 71 | 62 |  |
| **32** | 84 | 93 | 72 | 63 |  |
| **33** | 85 | 93 | 73 | 64 |  |
| **34** | 86 | 94 | 74 | 65 |  |
| **35** | 87 | 94 | 75 | 66 |  |
| **36** | 88 | 95 | 76 | 67 |  |
| **37** | 89 | 96 | 76 | 68 |  |
| **38** | 90 | 96 | 77 | 70 |  |
| **39** | 91 | 97 | 78 | 71 |  |
| **40** | 92 | 98 | 79 | 72 |  |
| **41** | 93 | 98 | 80 | 73 |  |
| **42** | 94 | 99 | 81 | 74 |  |
| **43** | 96 | 99 | 82 | 75 |  |
| **44** | 97 | 100 | 83 | 76 |  |
| **45** | 98 | 101 | 84 | 77 |  |
| **46** | 99 | 101 | 85 | 78 |  |
| **47** | 100 | 102 | 86 | 79 |  |
| **48** | 101 | 102 | 87 | 80 |  |
| **49** | 102 | 103 | 88 | 81 |  |
| **50** | 103 | 104 | 89 | 82 |  |
| **51** | 104 | 104 | 90 | 83 |  |
| **52** | 105 | 105 | 91 | 84 |  |
| **53** | 106 | 105 | 92 | 85 |  |
| **54** | 107 | 106 | 93 | 86 |  |
| **55** | 108 | 107 | 94 | 87 |  |
| **56** | 109 | 107 | 95 | 88 |  |
| **57** | 110 | 108 | 96 | 89 |  |
| **58** | 111 | 108 | 97 | 90 |  |
| **59** | 112 | 109 | 98 | 91 |  |
| **60** | 113 | 110 | 98 | 92 |  |
| **61** | 114 | 110 | 99 | 93 |  |
| **62** | 115 | 111 | 100 | 94 |  |
| **63** | 116 | 112 | 101 | 95 |  |
| **64** | 117 | 112 | 102 | 96 |  |
| **65** | 118 | 113 | 103 | 97 |  |
| **66** | 119 | 113 | 104 | 98 |  |
| **67** | 120 | 114 | 105 | 99 |  |
| **68** | 121 | 115 | 106 | 100 |  |
| **69** | 122 | 115 | 107 | 101 |  |
| **70** | 123 | 116 | 108 | 103 |  |
| **71** | 125 | 116 | 109 | 104 |  |
| **72** | 126 | 117 | 110 | 105 |  |
| **73** | 127 | 118 | 111 | 106 |  |
| **74** | 128 | 118 | 112 | 107 |  |
| **75** | 129 | 119 | 113 | 108 |  |
| **76** | 130 | 119 | 114 | 109 |  |
| **77** | 131 | 120 | 115 | 110 |  |
| **78** | 132 | 121 | 116 | 111 |  |
| **79** | 133 | 121 | 117 | 112 |  |
| **80** | 134 | 122 | 118 | 113 |  |
| **81** | 135 | 123 | 119 | 114 |  |
| **82** | 136 | 123 | 120 | 115 |  |
| **83** | 137 | 124 | 121 | 116 |  |
| **84** | 138 | 124 | 121 | 117 |  |
| **85** | 139 | 125 | 122 | 118 |  |
| **86** | 140 | 126 | 123 | 119 |  |
| **87** | 141 | 126 | 124 | 120 |  |
| **88** | 142 | 127 | 125 | 121 |  |
| **89** | 143 | 127 | 126 | 122 |  |
| **90** | 144 | 128 | 127 | 123 |  |
| **91** | 145 | 129 | 128 | 124 |  |
| **92** | 146 | 129 | 129 | 125 |  |
| **93** | 147 | 130 | 130 | 126 |  |
| **94** | 148 | 130 | 131 | 127 |  |
| **95** | 149 | 131 | 132 | 128 |  |
| **96** | 150 | 132 | 133 | 129 |  |
| **97** |  | 132 | 134 | 130 |  |
| **98** |  | 133 | 135 | 131 |  |
| **99** |  | 133 | 136 | 132 |  |
| **100** |  | 134 | 137 | 133 |  |
| **101** |  | 135 | 138 | 134 |  |
| **102** |  | 135 | 139 | 136 |  |
| **103** |  | 136 | 140 | 137 |  |
| **104** |  | 137 | 141 | 138 |  |
| **105** |  | 137 | 142 | 139 |  |
| **106** |  | 138 | 143 | 140 |  |
| **107** |  | 138 | 144 | 141 |  |
| **108** |  | 139 | 144 | 142 |  |
| **109** |  | 140 | 145 | 143 |  |
| **110** |  | 140 | 146 | 144 |  |
| **111** |  | 141 | 147 | 145 |  |
| **112** |  | 141 | 148 | 146 |  |
| **113** |  | 142 | 149 | 147 |  |
| **114** |  | 143 | 150 | 148 |  |
| **115** |  | 143 |  | 149 |  |
| **116** |  | 144 |  | 150 |  |
| **117** |  | 144 |  |  |  |
| **118** |  | 145 |  |  |  |
| **119** |  | 146 |  |  |  |
| **120** |  | 146 |  |  |  |
| **121** |  | 147 |  |  |  |
| **122** |  | 148 |  |  |  |
| **123** |  | 148 |  |  |  |
| **124** |  | 149 |  |  |  |
| **125** |  | 149 |  |  |  |
| **126** |  | 150 |  |  |  |
