# Supplementary material for: Validation of a Motor Competence Assessment Tool for Children and Adolescents (KTK3+) With Normative Values for 6- to 19-Year-Olds
Source: Front Physiol. 2021 Jun 23;12:652952. doi: 10.3389/fphys.2021.652952 (PMC8260948; doi:10.3389/fphys.2021.652952)
Supplement: Supplementary file 1 [file Data_Sheet_1.ZIP › Supplementary_Material_Table_D.docx]

Supplementary Material

| **Table D.** Motor Quotient (MQ) scores. MQ1 = MQ for Jumping Sideways ; MQ2 = MQ for Moving Sideways ; MQ3 = MQ for Balancing Backwards ; MQ4 = MQ for Eye-Hand Coordination ; M-Q = MQ for KTK3+ . | | | | | | | | |
| --- | --- | --- | --- | --- | --- | --- | --- | --- |
| **Sum MQ1-MQ4** | **M-Q** |  | **Sum MQ1-MQ4** | **M-Q** |  | **Sum MQ1-MQ4** | **M-Q** |  |
| 160-162 | 40 |  | 319-322 | 80 |  | 478-481 | 120 |  |
| 162-166 | 41 |  | 323-325 | 81 |  | 482-485 | 121 |  |
| 167-170 | 42 |  | 326-329 | 82 |  | 486-490 | 122 |  |
| 171-174 | 43 |  | 330-333 | 83 |  | 491-494 | 123 |  |
| 175-177 | 44 |  | 334-338 | 84 |  | 495-497 | 124 |  |
| 178-181 | 45 |  | 339-342 | 85 |  | 498-501 | 125 |  |
| 182-185 | 46 |  | 343-346 | 86 |  | 502-505 | 126 |  |
| 187-190 | 47 |  | 347-349 | 87 |  | 506-510 | 127 |  |
| 191-194 | 48 |  | 350-353 | 88 |  | 511-514 | 128 |  |
| 195-197 | 49 |  | 354-358 | 89 |  | 515-517 | 129 |  |
| 198-201 | 50 |  | 359-362 | 90 |  | 518-521 | 130 |  |
| 202-205 | 51 |  | 363-366 | 91 |  | 522-525 | 131 |  |
| 206-210 | 52 |  | 367-369 | 92 |  | 526-530 | 132 |  |
| 211-214 | 53 |  | 370-373 | 93 |  | 531-534 | 133 |  |
| 215-217 | 54 |  | 374-377 | 94 |  | 535-538 | 134 |  |
| 218-221 | 55 |  | 378-382 | 95 |  | 539-541 | 135 |  |
| 222-225 | 56 |  | 383-386 | 96 |  | 542-545 | 136 |  |
| 226-230 | 57 |  | 387-389 | 97 |  | 546-549 | 137 |  |
| 231-234 | 58 |  | 390-393 | 98 |  | 551-554 | 138 |  |
| 235-238 | 59 |  | 394-397 | 99 |  | 555-558 | 139 |  |
| 239-241 | 60 |  | 398-402 | 100 |  | 559-561 | 140 |  |
| 242-245 | 61 |  | 403-406 | 101 |  | 562-565 | 141 |  |
| 246-249 | 62 |  | 407-410 | 102 |  | 566-569 | 142 |  |
| 251-254 | 63 |  | 411-413 | 103 |  | 570-573 | 143 |  |
| 255-258 | 64 |  | 414-417 | 104 |  | 574-578 | 144 |  |
| 259-261 | 65 |  | 418-422 | 105 |  | 579-582 | 145 |  |
| 262-265 | 66 |  | 423-426 | 106 |  | 583-585 | 146 |  |
| 266-269 | 67 |  | 427-430 | 107 |  | 586-589 | 147 |  |
| 270-274 | 68 |  | 431-433 | 108 |  | 590-594 | 148 |  |
| 275-278 | 69 |  | 434-437 | 109 |  | 595-598 | 149 |  |
| 279-282 | 70 |  | 438-441 | 110 |  | 599-602 | 150 |  |
| 283-285 | 71 |  | 442-446 | 111 |  |  |  |  |
| 286-289 | 72 |  | 447-450 | 112 |  |  |  |  |
| 290-294 | 73 |  | 451-453 | 113 |  |  |  |  |
| 295-298 | 74 |  | 454-457 | 114 |  |  |  |  |
| 299-302 | 75 |  | 458-461 | 115 |  |  |  |  |
| 303-305 | 76 |  | 462-466 | 116 |  |  |  |  |
| 306-309 | 77 |  | 467-470 | 117 |  |  |  |  |
| 310-313 | 78 |  | 471-474 | 118 |  |  |  |  |
| 315-318 | 79 |  | 475-477 | 119 |  |  |  |  |
