# Supplementary material for: Validation of a Motor Competence Assessment Tool for Children and Adolescents (KTK3+) With Normative Values for 6- to 19-Year-Olds
Source: Front Physiol. 2021 Jun 23;12:652952. doi: 10.3389/fphys.2021.652952 (PMC8260948; doi:10.3389/fphys.2021.652952)
Supplement: Supplementary file 1 [file Data_Sheet_1.ZIP › Supplementary_Material_Table_E.docx]

| **Table E.** Classification M-Q | |  | |  | |  |
| --- | --- | --- | --- | --- | --- | --- |
| **M-Q** | **Classification** | | **Percentile** | | **Share in %** | |
| **131-145** | High gross MC proficiency | | 99-100 | | 2 | |
| **116-130** | Hood gross MC proficiency | | 85-98 | | 14 | |
| **86-115** | Normal gross MC proficiency | | 14-84 | | 68 | |
| **71-85** | Moderate gross MC disorder | | 3-16 | | 14 | |
| **56-70** | Severe gross MC disorder | | 0-2 | | 2 | |

Supplementary Material
